# Glia-derived exosomal miR-274 targets Sprouty in trachea and synaptic boutons to modulate growth and responses to hypoxia

**DOI:** 10.1073/pnas.1902537116

**Published:** 2019-10-30

**Authors:** Yi-Wei Tsai, Hsin-Ho Sung, Jian-Chiuan Li, Chun-Yen Yeh, Pei-Yi Chen, Ying-Ju Cheng, Chun-Hong Chen, Yu-Chen Tsai, Cheng-Ting Chien

**Affiliations:** ^a^Institute of Molecular Biology, Academia Sinica, 11529 Taipei, Taiwan;; ^b^National Institute of Infectious Diseases and Vaccinology, National Health Research Institutes, 35053 Zhunan, Taiwan;; ^c^Taiwan International Graduate Program in Interdisciplinary Neuroscience, National Cheng Kung University and Academia Sinica, 11529 Taipei, Taiwan;; ^d^Institute of Neuroscience, National Yang-Ming University, 11221 Taipei, Taiwan;; ^e^Department of Life Science, Tunghai University, 40704 Taichung, Taiwan

**Keywords:** microRNA, glia, exosome, hypoxia, *Drosophila*

## Abstract

Secreted exosomal microRNAs (miRNAs) mediate interorgan/tissue communications by modulating target gene expression, thereby regulating developmental and physiological functions. However, the source, route, and function in target cells have not been formally established for specific miRNAs. Here, we show that glial miR-274 non-cell-autonomously modulates the growth of synaptic boutons and tracheal branches. Whereas the precursor form of miR-274 is expressed in glia, the mature form of miR-274 distributes broadly, including in synaptic boutons, muscle cells, and tracheal cells. Mature miR-274 is secreted from glia to the circulating hemolymph as an exosomal cargo, a process requiring ESCRT components in exosome biogenesis and Rab11 and Syx1A in exosome release. We further show that miR-274 can function in the neurons or tracheal cells to modulate the growth of synaptic boutons and tracheal branches, respectively. Also, miR-274 uptake into the target cells by AP-2–dependent mechanisms modulates target cell growth. In the target cells, miR-274 down-regulates Sprouty (Sty) through a targeting sequence at the *sty* 3′ untranslated region, thereby enhancing MAPK signaling and promoting cell growth. miR-274 expressed in glia of an *mir-274* null mutant is released as an exosomal cargo in the circulating hemolymph, and such glial-specific expression resets normal levels of Sty and MAPK signaling and modulates target cell growth. *mir-274* mutant larvae are hypersensitive to hypoxia, which is suppressed by miR-274 expression in glia or by increasing tracheal branches. Thus, glia-derived miR-274 coordinates growth of synaptic boutons and tracheal branches to modulate larval hypoxia responses.

## Significance

Our study provides significant advances in the understanding of circulating exosomal microRNA (miRNA) in animals. Circulating exosomal miRNAs mediate communication among tissues and organs. In glia, mature miR-274 is produced and secreted to the circulating hemolymph to target the recipient cells, neurons, and tracheal cells. We also identified the target gene *sty* whose expression is down-regulated by miR-274 in the target/recipient cells. Downregulation of Sty leads to upregulation of MAPK signaling, thereby promoting the growth of synaptic boutons and tracheal branches. Thus, glia-derived miR-274 might be deemed a “gliotransmitter” to mediate communication with neurons and tracheal cells. The modulation of tracheal branches by glial-derived miR-274 is also crucial for fine-tuning larval behavior in response to hypoxia.

Cells communicate at multiple levels during development, from short to long range, between the same or different types of cells, and between different tissues/organs in the body. Long-range communication requires transport of signals, leading to coordinated growth and differentiation in multicellular organisms. Several mechanisms for transporting long-range signals from source to target cells have been identified, including transport by extracellular vesicles (EVs) (1, 2). These EVs originate from at least 2 routes: direct shedding of plasma membranes to form microvesicles and secretion of intraluminal vesicles, or exosomes, from multivesicular bodies (MVBs). Exosomal transportation has been better characterized due to the consistent size of the vesicles (30 to 100 nm in diameter), easy detection in the circulatory system, and well-characterized cargoes (3). Furthermore, the physiological functions and diseases associated with secreted exosomes have been studied in greater detail (4).

Secreted exosomes host noncoding microRNAs (miRNAs) that functionally inhibit protein expression in the target or recipient cells (3). In animals, miRNAs are small RNAs of ∼22 nucleotides, which possess a seed region of typically 2 to 8 nucleotides at their 5′ ends that binds to sequences of the target messenger RNAs (mRNAs) to promote mRNA degradation or translational repression (5). Although cell-autonomous functions of miRNAs have been amply reported, non-cell-autonomous functions have only been recently discovered. Once secreted into the circulatory systems, miRNAs can target gene expression in distant tissues. During formation of immune synapses, exosomal miR-335 is transferred from T cells to antigen-presenting cells to down-regulate *SOX-4* mRNA translation (6). Exosomal miR-451 and miR-21 are transferred from glioblastoma to microglia to down-regulate *c-Myc* expression (7). Adipocyte-derived exosomal miR-99b down-regulates *Fgf21* mRNA and protein expressions in hepatic cells (8). In *Drosophila*, epithelial cells express *bantam* miRNA to regulate neuronal growth (9). miRNAs have also been isolated from the circulating hemolymph of *Drosophila* that could associate with exosomes to function systematically or in specific target cells (10). However, mechanistic links of different processes—such as the sources of exosomal miRNAs, their presence in circulating hemolymph, and their direct target genes in target cells, as well as functional modulation of recipient tissues and relevant physiological functions—have not been established for a specific miRNA, especially in a model organism that would greatly facilitate a clear mechanistic understanding at the genetic level.

During vertebrate development, formations of nerves and blood vessels share many cellular processes, including cone-like growth tips, branching patterns, and ramifying networks (11, 12). Pairs of signals and receptors such as Slit and Robo, Netrin and Unc5/DCC coreceptor, and Ephrin and Eph, which were identified as axon outgrowth regulators, have since been shown to regulate vasculogenesis (11, 12). Expression of vascular endothelial growth factor (VEGF), which plays critical roles in angioblast migration and vessel ingression, is spatiotemporally regulated in the neural tube during embryonic development (13). Although VEGF167 and the axon guidance signal Sema3A function separately in early vessel and nerve formation, both signals function through the shared receptor neurophilin-1 (14). During postdevelopmental stages, neuronal activity and oxygen delivery in the nervous system are prominently coupled, forming the neurovascular units (15). Given the extreme sensitivity of the nervous system to alterations of ions, nutrients, and potentially harmful molecules in the vascular system, an interface between both systems is necessary. Astrocytes in the mammalian brain that are structurally and functionally coupled to neuronal synapses and vascular endothelial cells directly regulate their activities and communications (16–20). The insect trachea, the prototypical vascular system, allows oxygen delivery to the inner parts of the animal body. Nerves, glial sheath, and tracheal branches have been described for the larval brains and adult neuromuscular junctions (NMJs) of *Drosophila* (21–23). Synapse organization and activity of larval NMJs, as well as their glial interactions, have also been well characterized (23–25).

We explored the coupling of synaptic boutons to tracheal branches at larval *Drosophila* NMJs, as a system for studying coordinated nervous and vascular development. We screened a collection of miRNA-knockout mutants and identified the *mir-274* mutant as having defects in both synaptic and tracheal growth. By fluorescent in situ hybridization (FISH), we showed that the miR-274 precursor was expressed in glia and the mature form was ubiquitously detected. Consistently, miR-274 was required in glia for synaptic and tracheal growth. Glial expression of miR-274 could be detected in the hemolymph of the larval circulatory system. Indeed, miR-274 was secreted as an exosomal cargo as shown by genetic analysis and biochemical fractionation. miR-274 targets the *sprouty* (*sty*) 3′ untranslated region (UTR) to down-regulate Sty expression, leading to enhancement of MAPK signaling and target cell growth. Intriguingly, the *mir-274* mutant with fewer tracheal branches was hypersensitive to hypoxia. Thus, glial miR-274 coordinates the growth of synaptic boutons and tracheal branches and links the developmental role to behavior responses to hypoxia.

## Results

### miR-274 Is Required in Glia to Modulate Synaptic and Tracheal Growth.

**Fig. 1.**
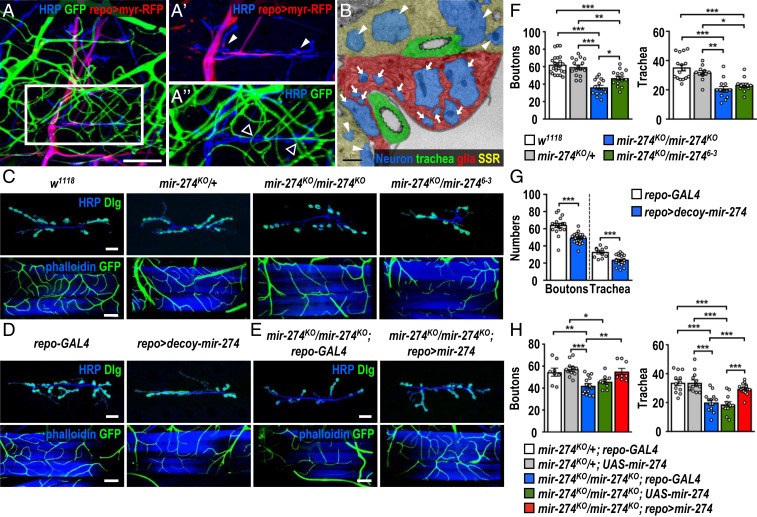
Reduced synaptic boutons and tracheal branches in miR-274 mutants. (*A* to *A*″) Confocal images show the glia–neuron–trachea system at NMJs of muscle 6/7 for screening miRNA mutants. Larvae were dissected to view glia labeled by *repo-GAL4*>*UAS-myr-RFP*, trachea labeled by *btl-lexA > lexAop-CD2-GFP*, and nerves stained by horseradish peroxidase (HRP). (Scale bar: 30 µm.) Boxed area in *A* is amplified to show that (*A*′) glial processes wrap around motor axons but not synaptic boutons (white arrowheads), and (*A*″) tracheal branches localize close to synaptic boutons (empty arrowheads). (*B*) Transmission election microscopy micrograph shows that glial processes directly enwrap axons (arrows). Synaptic boutons (arrowheads) are recognized by synaptic vesicles and surrounding SSR. (Scale bar: 1 µm.) (*C*–*E*) Images show synaptic boutons (top rows, scale bars: 30 µm) immunostained for presynaptic HRP (blue) and postsynaptic Dlg (green) and tracheal branches (bottom rows, scale bars: 60 µm) revealed by *btl-lexA*>*lexAop-CD2-GFP* (green) counterstained for muscle phalloidin (blue), for (*C*) *w*^*1118*^, *mir-274*^*KO*^*/+*, *mir-274*^*KO*^*/mir-274*^*KO*^, and *mir-274*^*KO*^/*mir-274*^*6−3*^, (*D*) *repo-GAL4* control (crossed to *w*^*1118*^, *Left*), and *repo-GAL4* crossed to *UAS-decoy-mir-274* (*repo > decoy-mir274*, *Right*), and (*E*) the miR-274 mutant carrying *GAL4* control (*mir-274*^*KO*^/*mir-274*^*KO*^; *repo-GAL4*) and glial rescue (*mir-274*^*KO*^/*mir-274*^*KO*^; *repo > mir-274*). (*F*–*H*) Dotted bar graphs for quantification of synaptic boutons and tracheal branches. Data were analyzed by one-way ANOVA followed by Tukey post hoc (*F* and *H*) or independent *t* test (*G*). See *SI Appendix*, Table S2. *P < 0.05, **P < 0.01, and ***P < 0.001.

By immunostaining synaptic, glial, and tracheal structures, we show that glial processes wrap around incoming motor axons, and envelopment ends before terminal branching at muscle 6/7 (Fig. 1 *A* and *A*′, arrowheads). Axonal terminal branches form bouton-like structures that innervate muscles to form functional synapses. Prominently, multiple tracheal branches terminate near these synaptic boutons (Fig. 1*A*′′, empty arrowheads). Ultrastructure analysis by transmission electron microscopy shows that glial processes enwrap axonal processes close to tracheal branches (Fig. 1*B*, arrows). Synaptic boutons wrapped within the subsynaptic reticulum (SSR) are also visible (Fig. 1*B*, arrowheads). These observations suggest that the glia–synapse–trachea organization might represent functional units and its formation might be developmentally regulated.

To investigate whether these structures are developmentally coregulated, we screened 51 miRNA-knockout mutants (26) for defects in both synaptic boutons and tracheal branches. Only the mutant for miR-274 (*mir-274*^*KO*^/*mir-274*^*KO*^) displayed reduced growth of both synaptic boutons and tracheal branches (Fig. 1*C*). Quantification revealed that larvae homozygous for *mir-274*^*KO*^ exhibited about 40% reduction in the numbers of synaptic boutons and tracheal branches compared to both wild-type *w*^*1118*^ and *mir-274*^*KO*^*/+* larvae (Fig. 1*F*). We also detected reduced numbers of synaptic boutons and tracheal branches in the transheterozygous *mir-274*^*KO*^*/mir-274*^*6−3*^ mutant, confirming that the lack of miR-274 activity accounts for growth defects in both systems (Fig. 1 *C* and *F*). Interestingly, tracheal branches near the surrounding area of synaptic boutons were severely reduced (*SI Appendix*, Fig. S1 *A* and *B*). While the *mir-274*^*KO*^ mutant had reduced tracheal branches, the muscle area was equivalent to wild type, leading to the reduction in the overall density of branch tips (*SI Appendix*, Fig. S1*C*). We then quantified the distribution of branch tips in the bouton-surrounding proximal area, which accounts for about a quarter of the total muscle area (*SI Appendix*, Fig. S1 *A* and *B*). In wild type, the relative tracheal density within the proximal area was similar to that in the outer area (*SI Appendix*, Fig. S1*D*). In the *mir-274*^*KO*^ mutant, however, the relative tracheal density within the proximal area was markedly reduced as compared to that in the outer area (*SI Appendix*, Fig. S1*D*). Thus, miR-274 might play a role in recruiting tracheal branches to the synaptic bouton area. Furthermore, the reduction in synaptic boutons was not limited to NMJs of muscle 6/7, as we also observed synaptic bouton reduction at NMJs of muscle 4 in the *mir-274*^*KO*^ mutant (*SI Appendix*, Fig. S2 *A* and *B*). Likewise, tracheal branching was also compromised in the dorsal region of the *mir-274*^*KO*^ mutant (*SI Appendix*, Fig. S2 *A* and *B*). These data suggest that larvae lacking miR-274 fail to develop complete sets of synaptic boutons and tracheal branches.

To examine whether specific types of cells require miR-274 for growth of synaptic boutons and tracheal branches, we employed the *UAS-decoy-mir-274* transgene driven by cell-type-specific *GAL4* drivers to inhibit miR-274 functions. Neuronal *elav-GAL4*, glial *repo-GAL4*, and tracheal *btl-GAL4* were individually crossed to *UAS-decoy-mir-274* to analyze phenotypes in synaptic boutons and tracheal branches. Surprisingly, glial depletion caused significant reductions in synaptic boutons and tracheal branches (Fig. 1 *D* and *G*). However, miR-274 inhibition by the neuronal or tracheal driver had no obvious phenotypic impact (*SI Appendix*, Fig. S2 *C*–*F*). Whereas the glial processes at NMJs of muscle 6/7 presented a normal morphology in the *mir-274*^*KO*^ mutant, the hemolymph–brain barrier (HBB, which is mainly composed of glia) was defective (*SI Appendix*, Fig. S3 *A* and *B*), confirming the findings of a previous study (26). The defective HBB could be rescued with expression of miR-274 in glia, suggesting a cell-autonomous function in HBB formation or maintenance (*SI Appendix*, Fig. S3 *C* and *D*). The reductions in synaptic boutons and tracheal branches are not secondary to the defective HBB, as an intact HBB was retained in glial expression of *UAS-decoy-mir-274* (*SI Appendix*, Fig. S3 *E* and *F*) while synaptic boutons and tracheal branches were reduced (Fig. 1 *D* and *G*). Thus, it seems that glial inhibition of miR-274 is sufficient to compromise synaptic and tracheal growth.

Furthermore, glial expression of miR-274 is sufficient to restore synaptic and tracheal growth as described below. In the homozygous *mir-274*^*KO*^ mutant carrying either *repo-GAL4* or *UAS-mir-274* alone, the numbers of synaptic boutons and tracheal branches were fewer than the numbers in the heterozygous mutants carrying either *repo-GAL4* or *UAS-mir-274* alone (Fig. 1 *E* and *H*). In the homozygous *mir-274*^*KO*^ mutant carrying both *repo-GAL4* and *UAS-mir-274*, the numbers of synaptic boutons and tracheal branches were comparable to those in the heterozygous mutants (Fig. 1 *E* and *H*). These results strongly support that glia-expressed miR-274 promotes growth of synaptic boutons and tracheal branches.

### miR-274 Precursor Is Expressed in Glia and miR-274 Mature Form Is Detected Ubiquitously.

**Fig. 2.**
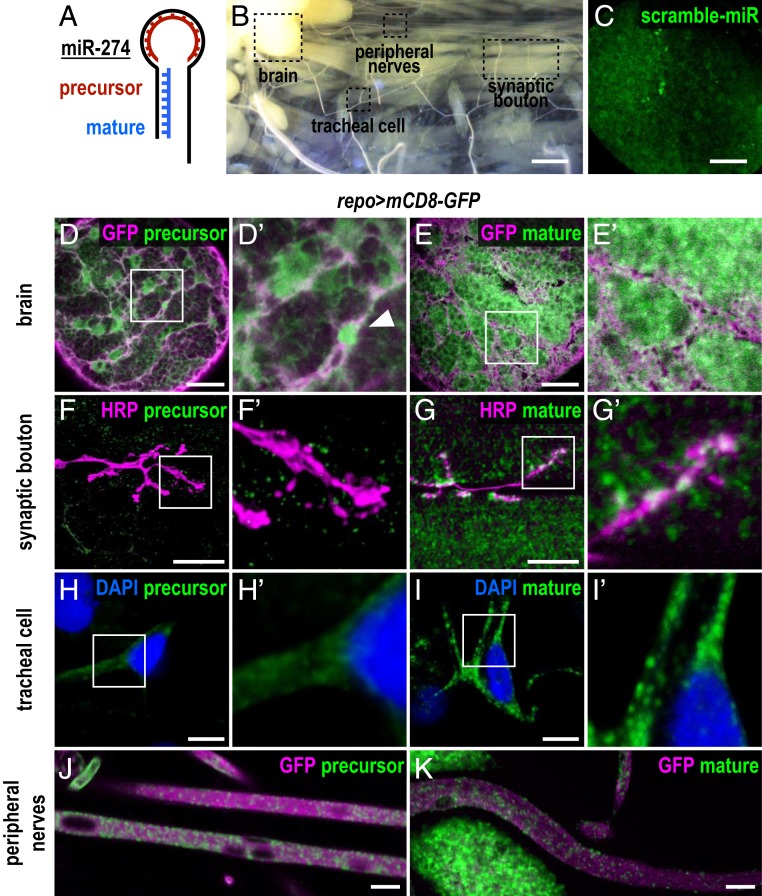
Expression of precursor and mature miR-274. (*A*) Illustration of the 2 FISH probes used to detect miR-274 expression. The precursor probe complements the loop region, and the mature probe complements the stem sequence. (*B*) Dissected third instar larvae (*w*^*1118*^) showing sites for detecting FISH signals in brain lobes, synaptic boutons, tracheal cells, and peripheral nerves. (Scale bar: 1 mm.) (*C*–*K*) Images for FISH signals (green) using the scramble (*C*), precursor (*D*, *F*, *H*, and *J*), or mature probe (*E*, *G*, *I*, and *K*), with glia labeled by *repo > mCD8-GFP* (magenta in *D*, *E*, *J*, and *K*), synaptic boutons by HRP (magenta in *F* and *G*), and DAPI-labeled nuclei (blue in *H* and *I*). (*C*) Low-background, nonspecific FISH signals detected by the scramble probe in the brain lobe. (Scale bar: 30 µm.) (*D*) The precursor probe detected a glial membrane pattern (enlarged image in *D*′) with a nucleus indicated by an arrowhead. (Scale bar: 30 µm.) (*F* and *H*) Only diffusive or low background signals were detected within synaptic boutons (*F* and *F*′) and tracheal cells (*H* and *H*′). (Scale bars: 10 µm.) (*E*, *G*, and *I*) The mature probe detected ubiquitous punctate signals in brain (*E* and *E*′, scale bar: 30 µm), synaptic boutons and muscles (*G* and *G*′, scale bar: 10 µm), and tracheal soma and branches (*I* and *I*′, scale bar: 10 µm). (*J* and *K*) Both precursor (*J*) and mature (*K*) miR-274 signals were observed in peripheral nerves. (Scale bars: 10 µm.)

To characterize miR-274 expression, we performed FISH experiments using probes complementary to the loop or the stem sequence to detect the precursor or the mature forms of miR-274 (Fig. 2*A*), respectively, in dissected larval fillets (Fig. 2*B*). In control using scrambled probes, we only detected low background or nonspecific signals in larval brain (Fig. 2*C*). However, the loop probe for detecting the miR-274 precursor presented prominent signals in the brain (Fig. 2 *D* and *D*′). These signals were localized in glia labeled with *repo-GAL4*–driven *mCD8-GFP* and occasionally strong nuclear signals were detected (Fig. 2*D*′, arrowhead). In contrast, low background signals were detected in synaptic boutons and tracheal branches (Fig. 2 *F*, *F*′, *H*, and *H*′). We then employed the stem probe to detect mature miR-274 (Fig. 2*A*). Interestingly, we observed strong and ubiquitous signals, that is, not restricted to specific cells in the brain (Fig. 2 *E* and *E*′). These signals were also detected in muscle cells and within synaptic boutons (Fig. 2 *G* and *G*′), as well as in tracheal soma and branches (Fig. 2 *I* and *I*′). These results suggest that the miR-274 precursor is mainly synthesized in glia and the mature form is detected in muscles, synaptic boutons, and tracheal cells.

Interestingly, both precursor and mature miR-274 signals were observed in peripheral nerves, suggesting a local peripheral regulation of synaptic boutons and tracheal branches (Fig. 2 *J* and *K*). To address the subtypes of peripheral glia in regulating synaptic and tracheal growth, subtype-specific *GAL4* drivers for perineurial (*NP6293-GAL4*), subperineurial (*moody-GAL4*), and wrapping (*Nrv2-GAL4*) glia were used to express *decoy-mir-274* for miR-274 trapping. The heterozygous *mir-274*^*KO*^*/+* background is more sensitive to *decoy-mir-274* trapping in glia (*SI Appendix*, Fig. S4 *A* and *B*), which was used in subtype glia trapping. Trapping miR-274 in pan glia led to significant growth defects (*SI Appendix*, Fig. S4 *A* and *B*), which was used in subtype glia trapping. Trapping miR-274 in subperineurial glia led to significant growth defects (*SI Appendix*, Fig. S4 *C* and *D*), while trapping miR-274 in perineural and wrapping glia had no effect (*SI Appendix*, Fig. S4 *E*–*H*). Thus, subperineurial glia might be the major source of miR-274 in regulating synaptic and tracheal growth.

### Exosomal Secretion of miR-274 from Glia Requires Rab11, Syx1A, and ESCRT Components.

**Fig. 3.**
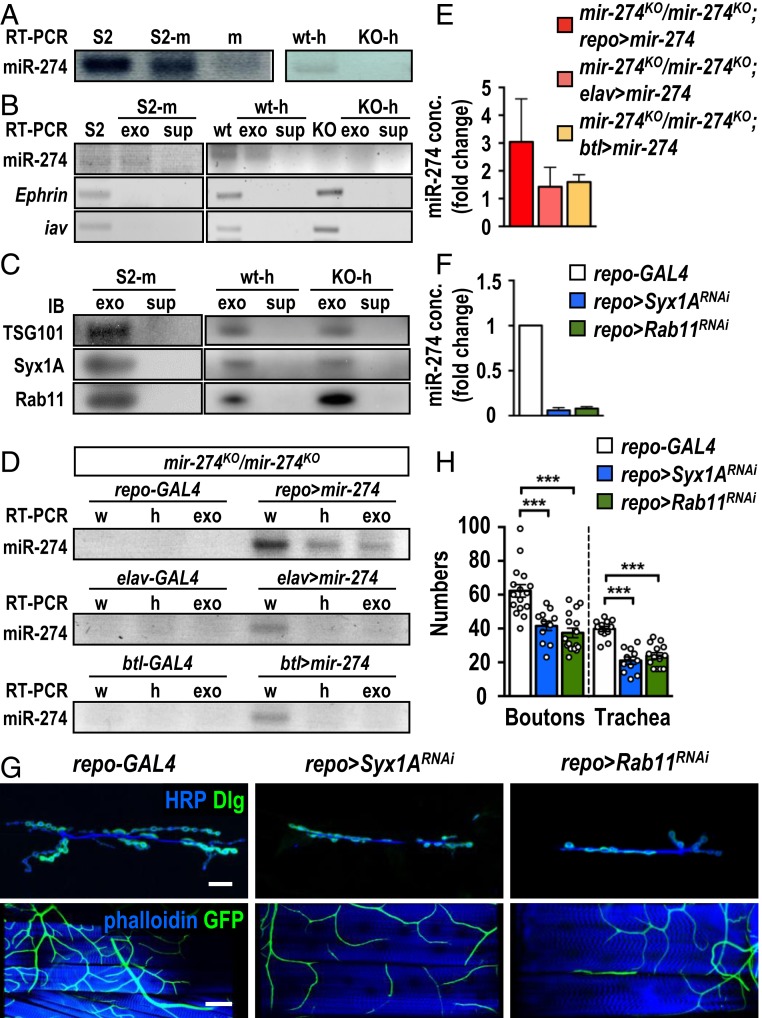
Secretion of exosomal miR-274 from glia. (*A*) Detection of miR-274 by RT-PCR in S2 cells (S2) and S2 cell-cultured medium (S2-m), and non-S2 cell-cultured medium (m) (*Left*). miR-274 was detected in *w*^*1118*^ hemolymph (wt-h) but not in *mir-274*^*KO*^ hemolymph (KO-h) (*Right*). (*B*) Detection of *mir-274*, *Ephrin*, and *iav* by RT-PCR in S2 cell lysates (S2), exosomal fractions (exo), and depleted supernatants (sup) from S2-medium (S2-m), *w*^*1118*^ larvae (wt) and *mir-274*^*KO*^ (KO) larvae. (*C*) Detection of TSG101, Syx1A, and Rab11 in Western blots for exosomal fractions (exo) and depleted supernatants (sup) from cultured S2 medium (S2-m), wild-type hemolymph (wt-h), and *mir-274*^*KO*^ hemolymph (KO-h). (*D*) Detection of miR-274 by RT-PCR in the *mir-274*^*KO*^ mutant with or without expression of *UAS-mir-274* by *repo-GAL4* (*Top*), *elav-GAL4* (*Middle*), or *btl-GAL4* (*Bottom*) in whole larval lysates (w), hemolymphs (h), and exosomal fractions (exo). (*E*) Quantification of miR-274 levels by absolute qPCR in larval exosomal fractions. (*F*) Absolute qPCR was performed to detect exosomal miR-274 levels in hemolymphs. (*G*) Confocal images of synaptic boutons (scale bar: 30 µm) and tracheal branches (scale bar: 60 µm) in *repo-GAL4* control, *repo > Syx1A*^*RNAi*^, and *repo > Rab11*^*RNAi*^. (*H*) Dotted bar graph for quantification of synaptic boutons and tracheal branches. See *SI Appendix*, Table S2. Data were analyzed by one-way ANOVA followed by Tukey post hoc. ***P < 0.001.

To examine how miR-274 is secreted, we first examined whether miR-274 could be secreted from S2 cells. Indeed, we detected miR-274 in S2 cell extracts (Fig. 3*A*). Significant levels of miR-274 could also be detected in the medium used to culture S2 cells, but not in the medium in which S2 cells were not cultured, indicating that miR-274 could be secreted from S2 cells into the medium (Fig. 3*A*). To examine whether secreted miRNA is through secretory exosomes, we isolated the exosomal fraction from the S2 cell culture medium. Indeed, miR-274 was detected in S2 cells and exosomal fractions from the conditioned medium (*SI Appendix*, Fig. S5*A*). As miR-274 might merely associate with the exosomes, the isolated exosomes with RNaseA treatment still included miR-274, suggesting that miR274 is likely enclosed inside the exosome (Fig. 3*B*). As negative control, *Ephrin* and *iav* mRNA that are not present in the secreted exosomes (27) were only detected in whole-cell extracts (Fig. 3*B*). As positive control, the exosomal proteins TSG101, Rab11, and Syntaxin1A (Syx1A) (28, 29) were detected in the exosomal fraction and not detected in the exosome-depleted supernatant (Fig. 3*C*).

As miRNAs could be transported by circulating exosomes, we then examined whether miR-274 could be detected in the larval hemolymph. Indeed, miR-274 was present in the hemolymph of wild-type larvae and absent in the hemolymph of the *mir-274*^*KO*^ mutant (Fig. 3*A*). We fractionated and pelleted exosomes from the hemolymph and found that fractionated exosomes were enriched with miR-274 (Fig. 3*B* and *SI Appendix*, Fig. S5*A*). However, the exosomal fraction isolated from the *mir-274*^*KO*^ hemolymph did not contain miR-274 (Fig. 3*B* and *SI Appendix*, Fig. S5*A*). Likewise, TSG101, Rab11, and Syx1A were detected in exosomal fractions of both wild-type and mutant larvae (Fig. 3*C*), and *Ephrin* and *iav* transcripts were absent in both exosomal fractions (Fig. 3*B*). Thus, miR-274 could be secreted into larval hemolymph and S2 cell culture medium as circulating exosomes.

With the detection of miR-274 in the exosomes of hemolymph, we would like to detect whether glia could secret miR-274 carried by exosomes into the hemolymph. As *mir-274*^*KO*^ is a deletion allele, no miR-274 could be detected in whole larval lysates, hemolymphs, and hemolymph-derived exosomal fractions in the homozygous *mir-274*^*KO*^ mutant (*mir-274*^*KO*^*/mir-274*^*KO*^; *repo-GAL4*; Fig. 3*D*). In contrast, miR-274 was detected in all 3 preparations from the homozygous *mir-274*^*KO*^ larvae carrying both *repo-GAL4* and *UAS-mir-274* (*mir-274*^*KO*^*/mir-274*^*KO*^; *repo > mir-274*; Fig. 3*D*). Thus, glia could secrete miR-274 as an exosomal cargo in the hemolymph. We performed the same set of experiments for neuronal or tracheal miR-274 expression in the *mir-274*^*KO*^ mutant. miR-274 was only detected in whole larval lysates but not in the isolated hemolymphs or exosomal fractions (Fig. 3*D*). Quantification of miR-274 levels in hemolymph-derived exosomal fractions showed a 3-fold increase in *mir-274*^*KO*^*/mir-274*^*KO*^; *repo > mir-274* as compared to *mir-274*^*KO*^*/mir-274*^*KO*^; *repo-GAL4*, whereas *elav-GAL4-* and *btl-GAL4-*driven expressions were slightly increased when compared to respective *GAL4* driver controls (Fig. 3*E*). Therefore, glia is the major type of cells that release exosomal miR-274 into the hemolymph.

Exosomal release requires Rab11 in MVB transportation and Syx1A in membrane fusion with the plasma membrane (30). To show that glial secretion of miR-274 requires Rab11 and Syx1A, we performed glial knockdown by *repo-GAL4*–driven *Rab11*^*RNAi*^ or *Syx1A*^*RNAi*^ expression. In both knockdowns the levels of the miR-274 transcript were dramatically reduced in hemolymph-isolated exosomal fractions, further confirming that glia is the major source for miR-274 expression and secretion (Fig. 3*F*). We then examined whether the growth of synaptic boutons and tracheal branches was compromised by the inhibition of exosomal release. As expected, glial knockdowns of Rab11 or Syx1A caused reductions in synaptic and tracheal growth (Fig. 3 *G* and *H*). Thus, the lack of Rab11 or Syx1A in glia recapitulates the phenotypes of trapping miR-274 in glia. These results strongly support that the exosomal release pathway is essential for miR-274–carried exosomes to be released from glia.

Cargo-carrying exosomes are assembled through serial actions of the ESCRT complexes, which promote membrane invagination and formation of intraluminal vesicles in MVBs (29). To suggest that miR-274 is packaged as an exosomal cargo, we examined whether disruption of ESCRT complex components in glia could affect the level of circulating miR-274. We chose to knock down TSG101 of the ESCRT-I complex and Shrb of the ESCRT-III complex. *TSG101*^*RNAi*^ or *shrb*^*RNAi*^ knockdown in glia by *repo-GAL4* resulted in reduced levels of exosomal miR-274 in the hemolymph (*SI Appendix*, Fig. S5*B*) and efficient suppression of synaptic and tracheal growth (*SI Appendix*, Fig. S5 *C* and *D*). Taken together, these results are consistent with glial expression of miR-274 being secreted into the circulating hemolymph through the exosomal pathway to regulate synaptic and tracheal growth.

### Glia-Secreted miR-274 Is Present and Functions in Target Cells.

**Fig. 4.**
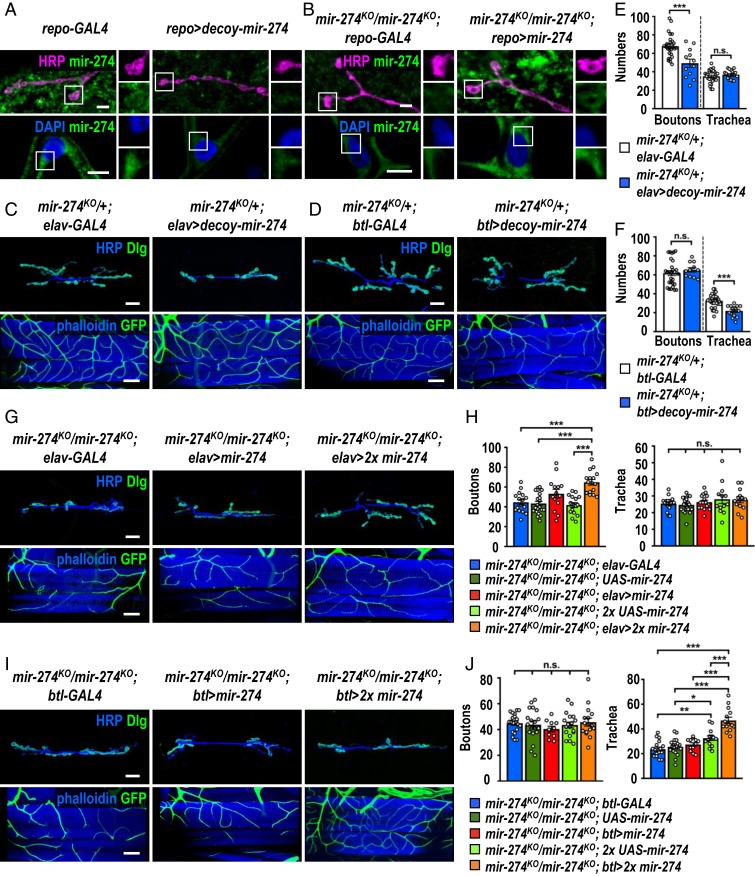
Glia-secreted miR-274 localizes and functions in target cells. (*A* and *B*) Images show FISH signals in synaptic boutons (*Top*, scale bars: 5 µm) and tracheal cells (*Bottom*, scale bar: 10 µm) for the mature miR-274 probe. Bright punctate signals were detected in *repo-GAL4* control (*A*, *Left*) and the glial rescue *mir-274*^*KO*^*/mir-274*^*KO*^*; repo > mir-274* (*B*, *Right*), whereas low background signals were presented in glial miR-274 trapping *repo > decoy-mir-274* (*A*, *Right*) and mutant control *mir-274*^*KO*^*/mir-274*^*KO*^*; repo-GAL4* (*B*, *Left*). Boxed areas are enlarged at right. (*C*, *D*, *G*, and *I*) Confocal images show phenotypes in growth of synaptic boutons (*Top*, scale bars: 30 µm) and tracheal branches (*Bottom*, scale bars: 60 µm). (*C* and *D*) Restricted miR-274 trapping in *elav-GAL4*-expressing neurons (*C*, *Right*) and *btl-GAL4*-expressing trachea (*D*, *Right*) and controls without the trapping *decoy-mir-274* transgene (*Left*). (*G* and *I*) Neuronal rescues by *elav-GAL4* (*G*) and tracheal rescues by *btl-GAL4* (*I*) drive 1 copy (*Middle*) or 2 copies (*Right*) of *UAS-mir-274*, compared to *GAL4* drivers control without rescuing transgene (*Left*). (*E*, *F*, *H*, and *J*) Dotted bar graphs for quantification of synaptic boutons and tracheal branches. See *SI Appendix*, Table S2. Data were analyzed by independent *t* test (*E* and *F*) or one-way ANOVA followed by Tukey post hoc (*H* and *J*). n.s., no significance; *P < 0.05, **P < 0.01, and ***P < 0.001.

To further show that glia-expressed miR-274 could reach synaptic boutons and tracheal branches for function, we first performed the FISH experiment with the mature miR-274 probe. Whereas the FISH signals were detected in synaptic boutons, muscle, and tracheal cells in the *repo-GAL4* control, low background signals were present in these structures by glial trapping of miR-274 (Fig. 4*A*). Also, while the *mir-274*^*KO*^ null mutant carrying only *repo-GAL4* (*mir-274*^*KO*^*/mir-274*^*KO*^; *repo-GAL4*) showed low background FISH signals throughout different tissues, glial expression of miR-274 in the *mir-274*^*KO*^ null mutant (*mir-274*^*KO*^*/mir-274*^*KO*^; *repo > mir-274*) displayed strong and punctate signals in synaptic boutons, muscle cells, and tracheal cells (Fig. 4*B*). We also examined neuronal expression of *UAS-mir-274* in the *mir-274*^*KO*^ mutant, which showed strong miR-274 FISH signals in synaptic boutons and muscle cells and low background signals in tracheal cells (*SI Appendix*, Fig. S6*A*). In contrast, tracheal *UAS-mir-274* expression in the *mir-274*^*KO*^ mutant showed strong miR-274 FISH signals in tracheal cells but not in synaptic boutons and muscle cells (*SI Appendix*, Fig. S6*B*). We also failed to detect the FISH signals in both synaptic boutons and tracheal cells upon glial knockdown of Rab11 or Syx1A (*SI Appendix*, Fig. S6*C*). Taken together, these results are consistent with the idea that only miR-274 expressed in glia could reach all target cells.

Glial expression of miR-274 localized in synaptic boutons and tracheal cells (Fig. 4*B*) and rescued their growth (Fig. 1 *E* and *H*), suggesting that miR-274 could function directly in target cells. To test this hypothesis, 3 different strategies were employed. First, we trapped miR-274 in target cells to disrupt miR-274 function in growth promotion. Previously, we showed that trapping miR-274 in the target cells by expressing the decoy transgene failed to recapitulate respective phenotypes (*SI Appendix*, Fig. S2 *C*–*F*), which we suspected might be due to insufficient miR-274 trapping. We therefore expressed the *UAS-decoy-mir-274* transgene in the *mir-274* heterozygous mutant that reduces one gene dosage of *mir-274*. Strikingly, trapping miR-274 in glia in *mir-274*^*KO*^*/+* caused stronger reductions in synaptic and tracheal growth than in the wild-type background (compare *SI Appendix*, Fig. S4 *A* and *B* to Fig. 1 *D* and *G*). Trapping miR-274 in neurons caused specific reduction in synaptic boutons (Fig. 4 *C* and *E*), and trapping miR-274 in tracheal cells caused specific reduction in tracheal branches (Fig. 4 *D* and *F*). In both cases, target cells without expressing the trapping *UAS-decoy-mir-274* transgene had no growth deficit (Fig. 4 *C*–*F*). Second, we expressed miR-274 in target cells for rescuing growth in the *mir-274*^*KO*^ mutant. We observed slight but nonsignificant rescuing effects when the *UAS-mir-274* transgene was expressed in target cells in the *mir-274*^*KO*^ mutant (Fig. 4 *G*–*J*). We argue that the levels of miR-274 expression might be insufficient. Therefore, 2 copies of the *UAS-mir-274* transgenes were used to express higher levels of miR-274 in the rescuing experiment. Consistently, neuronal expression of miR-274 in the *mir-274*^*KO*^ mutant restored synaptic boutons but not tracheal branches (Fig. 4 *G* and *H*), and tracheal expression of miR-274 restored the branch number only (Fig. 4 *I* and *J*). Thus, the specific rescuing results also support that miR-274 could function directly in target cells. Third, we disrupted miR-274 uptake in target cells to recapitulate *mir-274*^*KO*^ mutant phenotypes. EVs are endocytosed into recipient cells through the clathrin-dependent machinery (31). By expressing *AP-2α*^*RNAi*^ to block endocytosis in motor neurons (*D42-GAL4*) or tracheal cells (*btl-GAL4*), the miR-274 FISH signals were reduced in synaptic boutons or tracheal cells, respectively, suggesting that miR-274 failed to be endocytosed into the target cells (*SI Appendix*, Fig. S7 *A* and *B*). Consistently, specific reduction in growth of synaptic boutons or tracheal branches was observed (*SI Appendix*, Fig. S7 *C*–*F*). Taken together, these results strongly suggest that miR-274 is necessary and sufficient to function in target cells to regulate their growth.

### *sty* Is a Target Gene of miR-274 to Regulate Synaptic and Tracheal Growth.

**Fig. 5.**
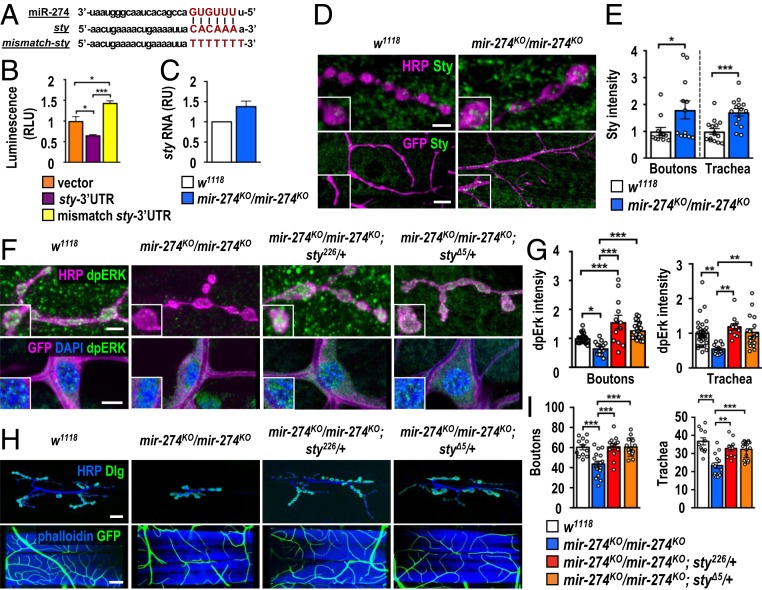
Regulation of Sty and dpERK expressions by miR-274 in synaptic boutons and tracheal cells. (*A*) Sequences show miR-274 with seeding sequence in red, the miR-274 targeting sequence at the *sty* 3′ UTR in red, and the mutated mismatch sequence in red. (*B*) Luciferase reporter assay for vector control, *sty* 3′ UTR, and mismatch 3′ UTR. Relative luciferase units (RLUs) were calculated from normalization to an internal control. (*C*) The *sty* transcript levels were measured by qPCR and normalized to *Rpl19*, with the relative unit (RU) of *w*^*1118*^ set as 1. (*D*) Images showing Sty immunostaining in synaptic boutons (scale bar: 5 µm) and tracheal branches (scale bar: 15 µm) in *w*^*1118*^ and *mir-274*^*KO*^*/mir-274*^*KO*^. (*F*) Images for dpERK immunostaining in synaptic boutons (scale bar: 5 µm) and tracheal cells (scale bar: 10 µm) in *w*^*1118*^, *mir-274*^*KO*^*/mir-274*^*KO*^, and *sty* suppression in *mir-274*^*KO*^*/mir-274*^*KO*^*; sty*^*226*^*/+* and *mir-274*^*KO*^*/mir-274*^*KO*^*; sty*^*Δ5*^*/+*. Boxed areas are enlarged images. (*H*) Images show the phenotypes of synaptic boutons (scale bar: 30 µm) and tracheal branches (scale bar: 60 µm). (*E*, *G*, and *I*) Dotted bar graphs for quantifications of Sty (*E*) and dpERK (*G*) immunofluorescence intensities within synaptic boutons and tracheal cells, and numbers of synaptic boutons and tracheal branches (*I*). See *SI Appendix*, Table S2. Data were analyzed by one-way ANOVA followed by Tukey post hoc tests (*B*, *G*, and *I*) or independent *t* test (*E*). *P < 0.05, **P < 0.01, and ***P < 0.001.

To understand how miR-274 regulates synaptic and tracheal growth, we searched for genes that harbor miR-274 target sites and exhibited up-regulation in the *mir-274*^*KO*^ larvae (*SI Appendix*, Fig. S8 *A* and *B*). From the candidate genes, we chose *sprouty* (*sty*) for further study since Sty plays a critical role in feedback inhibition of receptor tyrosine kinase (RTK)/MAPK signaling during tracheal branching and synaptic bouton formation (32–35). The 3′ UTR of *sty* mRNA contains a target site for miR-274 recognition. We then generated 2 luciferase reporter transgenes carrying the *sty* 3′ UTR with either precise or mismatched miR-274 targeting sequences (Fig. 5*A*). As expected, the precise miR-274 targeting sequence down-regulated reporter activity (relative to the vector control) when it was cotransfected with miR-274 (Fig. 5*B*). The mismatched reporter was not down-regulated upon miR-274 cotransfection (Fig. 5*B*). Thus, the *sty* mRNA level might be regulated by miR-274 through its 3′ UTR targeting sequence. We then addressed whether miR-274 regulates *sty* mRNA expression in vivo. Indeed, higher *sty* transcript levels were detected in *mir-274*^*KO*^ larvae compared to the levels in the wild-type control, consistent with miR-274’s having a role in downregulating *sty* expression (Fig. 5*C*).

We further confirmed that Sty is regulated by miR-274 in synaptic boutons and tracheal branches by performing immunostaining. We detected low levels of Sty expressions in synaptic boutons and tracheal branches in the wild-type control (Fig. 5*D*). In the *mir-274*^*KO*^ mutant, the levels of Sty were enhanced, which is supported by quantifications of Sty immunofluorescence intensities (Fig. 5 *D* and *E*). Sty expression was also up-regulated in muscle cells, suggesting that miR-274 might exert systemic regulation in multiple tissues (Fig. 5*D*). As a negative regulator, Sty inhibits several downstream components in RTK/MAPK signaling, leading to down-regulation of MAPK activity and inhibition of tissue growth (32–35). We further examined MAPK signaling activity by performing diphosphorylated-ERK (dpERK) immunostaining (35). Levels of dpERK in synaptic boutons and tracheal cells of *mir-274*^*KO*^ were reduced as compared to wild-type controls (Fig. 5 *F* and *G*). Down-regulation of dpERK levels depends on Sty, as elimination of one copy of *sty* in *mir-274*^*KO*^ (*mir-274*^*KO*^*/mir-274*^*KO*^; *sty*^*226*^*/+* or *mir-274*^*KO*^*/mir-274*^*KO*^; *sty*^*Δ5*^*/+*) restored dpERK levels in tracheal cells and synaptic boutons to the levels comparable to the control (Fig. 5 *F* and *G*). Restoration of dpERK levels was also detected in muscle (Fig. 5*F*). We then examined whether miR-274 negatively regulates Sty expression to modulate synaptic and tracheal growth. Indeed, reducing the *sty* gene dosage in the miR-274 mutant suppressed both growth phenotypes (Fig. 5 *H* and *I*). These data support that miR-274 inhibits Sty expression, which leads to MAPK activation to promote the growth of synaptic boutons and tracheal branches.

**Fig. 6.**
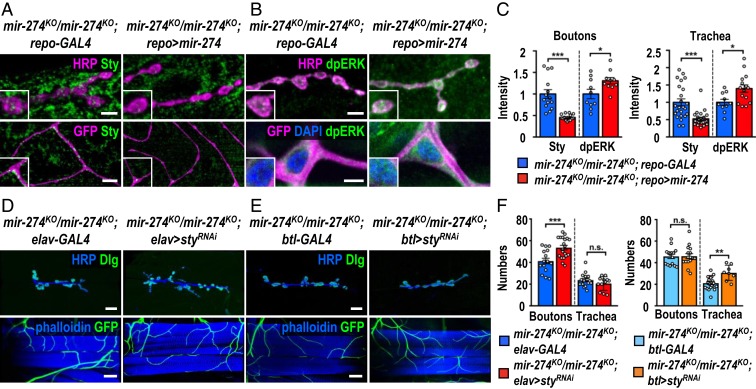
Glia-derived exosomal miR-274 modulates synaptic and tracheal growth. (*A* and *B*) Confocal images show immunostaining of Sty (*A*) or dpERK (*B*) in synaptic boutons (scale bars: 5 µm) and tracheal cells (scale bars: 15 µm in *A* and 10 µm in *B*) in *mir-274*^*KO*^*/mir-274*^*KO*^; *repo-GAL4* and *mir-274*^*KO*^*/mir-274*^*KO*^; *repo > mir-274*. Boxed areas are enlarged images. (*D* and *E*) Images show specific suppression of synaptic bouton growth in *mir-274*^*KO*^*/mir-274*^*KO*^; *elav > sty*^*RNAi*^, compared to *mir-274*^*KO*^*/mir-274*^*KO*^; *elav-GAL4* control (*D*), and specific growth suppression of tracheal branches in *mir-274*^*KO*^*/mir-274*^*KO*^; *btl > sty*^*RNAi*^, as compared to *mir-274*^*KO*^*/mir-274*^*KO*^; *btl-GAL4* (*E*). (Scale bars: 30 µm for synaptic boutons and 60 µm for tracheal branches.) (*C* and *F*) Dotted bar graphs for quantifications of Sty and dpERK immunofluorescence intensities within synaptic boutons and tracheal cells (*C*) and numbers of synaptic boutons and tracheal branches (*F*). See *SI Appendix*, Table S2. Data were analyzed by independent *t* tests. n.s., no significance; *P < 0.05, **P < 0.01, and ***P < 0.001.

To further show that glia-derived miR-274 regulates Sty and dpERK levels in neuronal and tracheal cells, we first performed immunostaining in glia-specific miR-274–rescued larvae that had restored synaptic and tracheal growth (Fig. 1 *E* and *H*). In glia-rescued larvae (*mir-274*^*KO*^*/mir-274*^*KO*^; *repo > mir-274*), we found reduced Sty and increased dpERK levels, as compared to those without glia-specific miR-274 rescue (*mir-274*^*KO*^*/mir-274*^*KO*^; *repo-GAL4*) (Fig. 6 *A*–*C*). Similarly, trapping miR-274 in glia induced higher Sty levels in synaptic boutons and tracheal branches compared to *repo-GAL4* (*SI Appendix*, Fig. S9 *A* and *B*). We also detected reduced levels of dpERK at these 2 sites (*SI Appendix*, Fig. S9 *C* and *D*). These results strongly support that glia-expressed miR-274 reaches target cells to down-regulate Sty expression and to promote growth of synaptic boutons and tracheal branches. We also examined the effects of disrupting exosomal biogenesis, transportation, and release in glia. By *repo-GAL4*–induced knockdowns of Rab11, Syx1A, TSG101 and Shrb, Sty up-regulation and dpERK down-regulation were detected in synaptic boutons and tracheal cells as well (*SI Appendix*, Fig. S9 *E*–*L*).

The *sty* mRNA was detected in exosomes isolated from S2 cells (ref. 27 and *SI Appendix*, Fig. S10). It is possible that *sty* was down-regulated and packaged in exosomes in glia for secretion. However, we failed to detect *sty* mRNA in the exosomes isolated from larval hemolymph, suggesting distinct populations of exosomes derived from S2 cells and larval hemolymph (*SI Appendix*, Fig. S10). Thus, *sty* is more likely to be directly down-regulated in target cells. To support this possibility, *sty* was specifically down-regulated in target cells of the *mir-274*^*KO*^ mutant, and the respective phenotype was analyzed. When *sty*^*RNAi*^ was expressed in neurons by *elav-GAL4* (*mir-274*^*KO*^*/mir-274*^*KO*^; *elav > sty*^*RNAi*^), growth of synaptic boutons but not that of tracheal branches was rescued (Fig. 6 *D* and *F*). Likewise, tracheal growth deficit was restored when the *sty*^*RNAi*^ transgene was driven by *btl-GAL4* (*mir-274*^*KO*^*/mir-274*^*KO*^; *btl > sty*^*RNAi*^) (Fig. 6 *E* and *F*). Taken together, these results suggest that glia-derived miR-274 down-regulates Sty and up-regulates dpERK levels in synaptic boutons and tracheal cells to regulate their growth.

### miR-274 Modulates Larval Hypoxia Response.

**Fig. 7.**
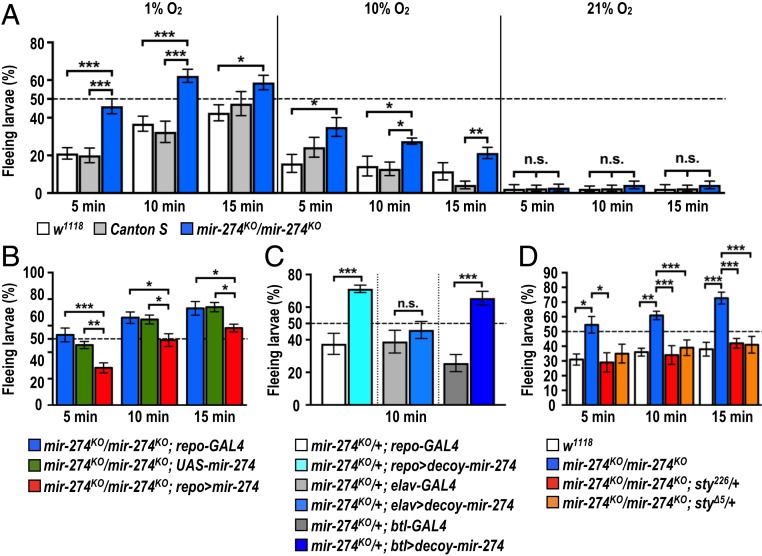
miR-274 modulates larval hypoxia responses. (*A*–*D*) Bar graphs show quantifications of percentages of larvae fleeing food sources under conditions of 1% (*A*, *Left* and *B*–*D*), 10% (*A*, *Middle*), or 21% (*A*, *Right*) oxygen. Data were analyzed by one-way ANOVA followed by Tukey post hoc test (*A*, *B*, and *D*) and independent *t* test (*C*). See *SI Appendix*, Table S2. n.s., no significance; *P < 0.05, **P < 0.01, and ***P < 0.001.

The *Drosophila* trachea is a highly branched network with open ends and air-filled terminal branches that function in gas exchange similarly to mammalian circulatory systems (36, 37). Terminal branching of trachea is induced by low oxygen tension (38). We postulated that miR-274–modulated tracheal branching might play a physiological role during hypoxia. To test this possibility, we assayed the larval escaping behavior in response to hypoxia (39). When exposed to hypoxia (1% O_2_), about 20% of control larvae (*w*^*1118*^ and *Canton S*) escaped away from the food paste within 5 min, and this percentage increased to almost 40% by 10 min and to close to 50% by 15 min (Fig. 7 *A*, *Left*). Strikingly, almost 50% of *mir-274*^*KO*^ mutants exhibited a strong hypoxia response by escaping away from the food paste by 5 min and about 60% by 10 and 15 min (Fig. 7 *A*, *Left*). The differential responses between *mir-274*^*KO*^ and control larvae were still significant when we conducted the assay in 10% O_2_, suggesting that the mutant larvae exhibited hypersensitivity toward reduced oxygen levels (Fig. 7 *A*, *Middle*). However, no significant differences were found when we assayed these 3 genotypes under normoxia, with almost all larvae (>95%) staying in the food source (Fig. 7 *A*, *Right*). We performed several control experiments to show that *mir-274*^*KO*^ larvae are indeed more responsive to lower oxygen levels. First, *mir-274*^*KO*^ mutants still exhibited a significantly different hypoxia response compared to control larvae in a 10-fold–diluted food source, suggesting that the enhanced fleeing behavior of mutant larvae is not due to differences in evaluating nutrition (*SI Appendix*, Fig. S11*A*). Second, the hypoxia-induced response is not caused by alterations in locomotion, as we observed comparable crawling lengths between *mir-274*^*KO*^ mutant and control larvae (*SI Appendix*, Fig. S11*B*). Third, feeding motivation toward nutritious (yeast) or nonnutritious (grape juice) foods, as evaluated by counting mouth hook contractions, were almost identical under both fed and starved conditions (*SI Appendix*, Fig. S11*C*). Fourth, we performed the behavior assay in response to high salt that serves as an alternative aversive stimulus (40). Similar to the hypoxia escape behavior, high-salt food induced larval fleeing behavior. We observed almost identical percentages of fleeing larvae between control and the *mir-274*^*KO*^ mutant. Thus, the *mir-274*^*KO*^ mutant seems respond specifically to hypoxia (*SI Appendix*, Fig. S11*D*).

We performed rescue experiments to examine whether glia-expressed miR-274 is required for normal larval hypoxia responses. Homozygous *mir-274*^*KO*^ mutants carrying both *repo-GAL4* and *UAS-mir-274* transgenes showed a reduced percentage of fleeing larvae in response to hypoxia compared to homozygous *mir-274*^*KO*^ mutants carrying either the *repo-GAL4* or *UAS-mir-274* transgenes (Fig. 7*B*). As glial rescue restored relative normal growth of synaptic boutons and tracheal branches (Fig. 1 *E* and *H*), we then examined specific types of cells that require miR-274 in the hypoxia response assay. Trapping miR-274 within glia (*mir-274*^*KO*^*/+*; *repo > decoy-mir-274*) significantly reduced synaptic boutons and tracheal branches (*SI Appendix*, Fig. S4 *A* and *B*). Consistently, trapping miR-274 within glia also induced the hyperactive hypoxia response (Fig. 7*C*). Intriguingly, the hyperactive hypoxia response was also observed in tracheal trapping in the *mir-274*^*KO*^*/+*; *btl > decoy-mir-274* larvae (Fig. 7*C*), which presented tracheal branch reduction (Fig. 4 *D* and *F*). However, neuronal trapping (*mir-274*^*KO*^*/+*; *elav > decoy-mir-274*) that reduced synaptic boutons but not tracheal branches (Fig. 4 *C* and *E*) exhibited normal hypoxia response (Fig. 7*C*). Since both glial and tracheal trapping caused defective tracheal ramification, these analyses suggest that the intact tracheal system is critical for normal hypoxia response. To examine whether miR-274 functions through Sty down-regulation to regulate the hypoxia response, genetic suppression was tested. Indeed, introducing a *sty* mutant allele in the homozygous *mir-274*^*KO*^ mutant (*mir-274*^*KO*^*/mir-274*^*KO*^; *sty*^*226*^*/+* or *mir-274*^*KO*^*/mir-274*^*KO*^; *sty*^*Δ5*^*/+*) almost completely suppressed the enhanced hypoxia escape response (Fig. 7*D*). Taken together, these results support that miR-274-regulated tracheal branching is linked to the hypoxia escape response.

## Discussion

We propose that miR-274–modulated synaptic and tracheal growth during development is coupled to physiological demands (*SI Appendix*, Fig. S12).

### Circulating miR-274.

Extracellular miRNAs detected in the blood serum and other body fluids are highly stable, making them ideal signaling molecules for long-distance communication among tissues and organs (41–44). *Drosophila* miRNAs are present in the circulatory system, likely to function systematically for tissue and organ interactions (10). We detected secreted miR-274–carrying exosomes from larval hemolymphs and S2 cultured media (Fig. 3 *A* and *B* and *SI Appendix*, Fig. S5*A*). Detection of miR-274–carrying exosomes in the hemolymph depends partially on the ESCRT components in exosome biogenesis (29) and Rab11 and Syx1A in exosome release (30) in glia (Fig. 3*F* and *SI Appendix*, Fig. S5*B*), supporting that glia-secreted miR-274 is carried by exosomes in the larval circulatory system. Circulating exosomes carry diverse molecules including proteins and RNAs. Although some studies have suggested that extracellular miRNAs might be “cellular by-products” disposed of by apoptotic cells (44), our genetic data provide strong evidence of a non-cell-autonomous developmental role for glial miR-274. Although miR-274 may cell-autonomously regulate secreted factors in glia to execute its function indirectly, our findings support an active and direct role for miR-274 in target cells. In addition to confirming the presence of miR-274 in target cells following glia-only expression (Fig. 4*B*), miR-274 trapping in glia also diminished its presence in target cells (Fig. 4*A*). We observed that Sty and dpERK expressions were regulated in target cells upon glia-only miR-274 expression (Fig. 6 *A* and *B*). Also, miR-274 trapping in target cells (Fig. 4 *C*–*F*) and target cell-specific rescue (Fig. 4 *G*–*J*) strongly support that miR-274 functions in target cells to regulate their growth. Circulating miR-274 in the hemolymph could potentially target multiple tissues given that we also detected miR-274 in muscle cells, whose function awaits further study. Accordingly, miR-274 might have a systematic role in multiple tissues, coordinating their developmental processes and postdevelopmental physiology.

### Glia Specificity of miR-274 Secretion.

The non-cell-autonomous role of miR-274 appears to be highly cell-type-specific. Although expression of precursor miR-274 is highly glia-enriched, perhaps accounting for the majority of specificity, other layers of regulation may confer this specificity. miR-274 secretion into the hemolymph is highly specific to glia, as only glia-expressed miR-274 was detected in synaptic boutons, muscle, and tracheal cells, whereas neuron- or trachea-expressed miR-274 was only detected in the respective expressing cells (Fig. 4*B* and *SI Appendix*, Fig. S6 *A* and *B*). Interestingly, neuron-expressed miR-274 was also detected in muscle cells (*SI Appendix*, Fig. S6*A*), which might be transported by transverse exosomes crossing the synaptic cleft at NMJs, similar to the Wnt/Wg signal carried by Evenness interrupted (Evi)-positive exosomes from pre- to postsynapses (30). Developmental signals like Hedgehog are also transported over long distances in wing epithelia for cell fate induction (28). The *Drosophila* retrovirus-like Gag protein Arc1 (dArc1) binds to *darc1* mRNA to be sorted into exosomes for transport across synaptic clefts (27). Interestingly, although presynaptic release of Wg/dArc1 and glial miR-274 shares a requirement for Rab11 and Syx1A, they may still exhibit substantial difference. Thus, multiple secretory exosomal pathways carry distinct cargos and function in different tissues of *Drosophila*.

Glia-specific miR-274 release suggests another layer of regulation for exosome-mediated cell–cell communication. Exosomes are formed through ubiquitination-dependent and -independent or ESCRT-dependent and -independent pathways that package different combinations of cargoes (2, 3). Neuronal or tracheal expressed miR-274 was not detected in hemolymph (Fig. 3*D*), perhaps because neurons or tracheal cells lack the specific pathways to generate miR-274–bearing exosomes. It has been suggested that miRNAs are subjected to modifications, including uridylation and adenylation that alter miRNA localization, stability, or activity (45). Such modifications may further induce packaging of miRNAs into exosomes for secretion in glia. Cargo packaging and exosome formation pathways are distinct in different types of cells (2, 3). We observed differential effects of knocking down several ESCRT components in terms of regulating synapse and tracheal growth, which could reflect the existence of heterogeneous populations of exosomes (*SI Appendix*, Fig. S5 *C* and *D*). Differential requirements for ESCRT components have also been observed for blocking Hh-borne exosomes from wing-disk epithelial cells (28), as well as in the presynaptic release of Evi-positive exosomes (30). Thus, it seems that complex regulation of the biogenesis of distinct exosomal populations may underlie exosome-mediated communications between specific pairs of source and target cells. Distinct miRNA species have been detected in exosomes isolated from various types of immune, cancer, adipose, and glial cells (6–8). Our analysis of the non-cell-autonomous function of miR-274 serves as a foundation for further study of the cell and tissue specificity involved in exosome-mediated cell–cell communication.

### Glia-Modulated Growth of Trachea Branches and Hypoxia Responses.

Similar to mammalian systems, *Drosophila* glia are linked to neurons and vascular systems in terms of their structure and function. In the larval *Drosophila* brain, trachea grow alongside glial processes toward the central neuropils (22). In the peripheral nervous system of adult flies, glial processes are intertwined with synaptic bouton-bearing axonal terminals and tracheal terminal branches to form functional complexes (21). This coupling between tracheal and neuronal processes may ensure efficient oxygen supply to neurons for activity and homeostasis, which is similar to the coupling between the vascular and nervous systems in vertebrates. At NMJs, the gliotransmitters Wnt/Wg and tumor necrosis factor-α regulate synaptic plasticity (46, 47). Glia also function as macrophages, engulfing synaptic debris and shaping neurites after injury (48). Direct ablation of glia throughout development induces tracheal branching, suggesting that tracheal branching is restricted by glia (22). In this study, we further report the coregulation of both tracheal and nervous systems by glial-derived miR-274, reinforcing the idea of glia–neurovascular coupling in *Drosophila*.

Here, we chose Sty to investigate miR-274 targeting since Sty is a negative regulator of RTK/Ras/MAPK signaling and is involved in synaptic growth and tracheal branching (32–35). Synaptic boutons are reduced when *sty* is overexpressed in neurons (32), a phenotype recapitulated in the *mir-274*^*KO*^ mutant. Loss-of-function mutations in *sty* enhanced tracheal branching (33). The glial regulation of Sty levels in 2 different types of cells could ensure synchronized growth regulation for both synaptic boutons and tracheal branches.

Recently, miRNAs were also shown to be essential for physiological functions. In *Drosophila*, miR-iab4/iab8 is expressed in self-righting node neurons (SRNs) controlling larval self-righting behavior. Lack of miR-iab4/iab8 or overexpressing the target gene *Ultrabithorax* in SRNs inhibits the ability of larvae to right themselves (49). Similarly, astrocyte-specific expression of miR-263b and miR-274 is essential for circadian locomotor activity rhythms (50). Here, we show that *mir-274*^*KO*^ mutants are sensitive to a sustained low-oxygen environment. This behavioral defect correlates with fewer tracheal branches. Oxygen may be delivered in the body less efficiently by having fewer tracheal terminal branches, rendering mutants less tolerant to low oxygen levels. Thus, miR-274 seems to ensure a well-developed tracheal system (and perhaps also synaptic boutons), allowing larvae to tolerate hypoxia. Our study highlights a coordinating role for glia in regulating a coupled developmental and physiological process.

## Materials and Methods

All materials (reagents and fly stocks; see *SI Appendix*) and protocols are available by contacting the corresponding author.

### Fly Stocks.

All flies were reared at 25 °C under a 12-h:12-h light:dark cycle. Third instar wandering larvae were used for experiments. See extended details in *SI Appendix*, *Materials and Methods*.

### Exosome Isolation.

Exosome fractions were isolated from the medium used for culturing *Drosophila* S2 cells (2 × 10^6^ cells per mL) or the hemolymph isolated from 50∼100 larvae. In brief, the S2 cell culture medium or larval extracellular fluid was centrifuged at the series of 300 × *g* for 5 min, 2,000 × *g* for 10 min, and 10,000 × *g* for 30 min to remove large cell debris. Exosomes were then collected following the manufacturer’s instructions for the ExoQuick kit (System Biosciences). For Fig. 3*B*, the isolated exosomes were further treated with 50 mg/mL RNaseA (Geneaid Biotech) for 30 min at 37 °C.

### Statistical Analysis.

Graphpad Prism v6 (Graphpad) was used to perform statistical analyses. All data are expressed as mean ± SEM.

See other extended methodological details in *SI Appendix*, *Materials and Methods*.

## Supplementary Material

Supplementary File
