# Supplementary material for: Glia-derived exosomal miR-274 targets Sprouty in trachea and synaptic boutons to modulate growth and responses to hypoxia
Source: Proc Natl Acad Sci U S A. 2019 Oct 30;116(49):24651–61. doi: 10.1073/pnas.1902537116 (PMC6900535; doi:10.1073/pnas.1902537116)
Supplement: Supplementary File [file pnas.1902537116.sapp.pdf]

PNAS
www.pnas.org

Supplementary Information for

Glia-derived exosomal miR-274 targets Sprouty in trachea and synaptic boutons to modulate growth and responses to hypoxia

Yi-Wei Tsai[1], Hsin-Ho Sung[1], Jian-Chiuan Li[2], Chun-Yen Yeh[1,3], Pei-Yi Chen[1,4], Ying-Ju Cheng[1], Chun-Hong Chen[2], Yu-Chen Tsai[5], Cheng-Ting Chien*[1,3,4]

[1]Institute of Molecular Biology, Academia Sinica, Taipei 11529, Taiwan

[2]National Institute of Infectious Diseases and Vaccinology, National Health Research Institutes, Zhunan 35053, Taiwan

[3]Taiwan International Graduate Program in Interdisciplinary Neuroscience, National Cheng Kung University and Academia Sinica, Taipei 11529, Taiwan

[4]Institute of Neuroscience, National Yang-Ming University, Taipei 11221, Taiwan

[5]Department of Life Science, Tunghai University, Taichung 40704, Taiwan

*Address correspondence to:

Cheng-Ting Chien, Ph.D
Email: ctchien@gate.sinica.edu.tw

Supplementary Information

Materials and Methods

Fly stocks

Mutant flies obtained from Bloomington Drosophila Stock Center (BDSC) include *mir-274*^{KO} (BDSC_58904), *sty*$^{\Delta5}$ (BDSC_6382) and *sty*^{226} (BDSC_6383). The *mir-274*$^{6\text{-}3}$ allele bears a mutation in the seeding motif. The GAL4/UAS expression system was used, with GAL4 driver lines: *repo-GAL4* (BDSC_7415), *elav-GAL4* (BDSC_458), *btl-GAL4* (BDSC_8807), *Nrv2-GAL4* (BDSC_6800), *moody-GAL4* (1), *NP6293-GAL4* (Kyoto Stock Center_105188) and *D42-GAL4* (BDSC_8816), and UAS responders lines: *UAS-mir-274* (BDSC_59895), *UAS-mCD8-GFP* (BDSC_5137) and *UAS-myr-RFP* (BDSC_7119). RNAi lines include *UAS-TSG101*^{RNAi} (BDSC_35710), *UAS-shrb*^{RNAi} (BDSC_38305), *UAS-Chmp1*^{RNAi} (BDSC_28906), *UAS-ALiX*^{RNAi} (BDSC_33417), *UAS-Rab11*^{RNAi} (BDSC_27730) and *UAS-Syx1A*^{RNAi} (BDSC_25811). *UAS-AP-2α*^{RNAi} (15565) and *UAS-Vps28*^{RNAi} (105124) were obtained from Vienna *Drosophila* Resource Center. The LexA/LexAop expression system was used in *btl-lexA*-driven *lexAop-CD2-GFP* to present the pattern of tracheal branches (2). The decoy-mir-274 construct was generated as described (3). In brief, decoy miR-274-binding sites were synthesized by PCR to assemble multiple copies of the decoy-linker-mir-274 cassette. Three copies of the decoy-linker-mir-274 cassette were integrated into the pUAST vector (*Drosophila* Genomics Resource Center) to generate the decoy-mir-274 plasmid for producing transgenic lines.

Immunostaining, microscopy and image processing

Dissected third instar larval fillets were fixed and stained as described. Z-stack confocal images were obtained by Zeiss LSM510 or LSM 710 microscopy and processed by Imaris 8.4.1 (Bitplane). Primary antibodies used in this study were goat anti-HRP-conjugated TRITC (1:100; Jackson Lab), rabbit anti-HRP-conjugated 647 (1:100; Jackson Lab), chicken anti-GFP (1:500; Abcam), mouse anti-Dlg (1:100; Developmental Studies Hybridoma Bank, DSHB), mouse anti-activated MAP kinase (diphosphorylated ERK-1&2, 1:20; Sigma-Aldrich), and rabbit anti-Sty (1:50) (4). Muscles were visualized by TRITC-conjugated phalloidin (1:2,000; Sigma-Aldrich). Tracheal nuclei were visualized by DAPI (1:5,000; Sigma-Aldrich). Goat anti-chicken (Invitrogen) or mouse Alexa Fluor 488- (Jackson Lab), goat anti-rabbit Cy5- (Jackson Lab) and goat anti-mouse Cy5-conjugated (Jackson Lab) secondary antibodies were used at 1:500 dilution. Signal intensities of Sty in synaptic boutons and dpERK in tracheal

branches or cell nuclei were quantified using Fiji image processing package (https://fiji.sc/). In brief, fluorescent integrated densities of Sty or dpERK were measured within HRP-labeled synaptic boutons, GFP-labeled tracheal branches, or DAPI-labeled tracheal nuclei. The integrated densities were normalized to background readings of respective images. For electron microscopy imaging, ultrastructures of dissected third instar larvae were processed as described to reveal glia-neuron-trachea organization (5).

Fluorescent *in situ* hybridization (FISH)

To detect precursor miR-274, we designed a 5'-biotin-labeled probe complementary to the loop sequence. To design a probe for mature miR-274 and a scramble probe, we used a 5'-DIG-labeled miRCURY LNA probe complementary (or scrambled) to the stem sequence (Exiqon). Wandering larvae were dissected to perform EDC-fixed FISH as described (6-8). After hybridization, samples were blocked in Odyssey Blocking Buffers/PBT (1:1) solution (LI-COR Biosciences) for 1 hr and incubated overnight with sheep anti-DIG-POD primary antibody (1:1,000; Roche) or mouse anti-biotin (1:1,000; Abcam) in the WB:PBT solution at 4°C. Samples with the biotinylated probe were incubated for 2 hr in biotinylated donkey anti-mouse secondary antibody (1:500; Jackson Lab) in Odyssey Blocking Buffers/PBT solution, washed three times in PBS for 10 min, and then incubated in HRP-labeled avidin-biotin complex (ABC; Vector Laboratories) solution for 1 hr. After washing three times in PBT with 10 min each, all samples were incubated in TSA Plus Cyanine5 Evaluation kit (1:50; PerkinElmer) for signal amplification at room temperature in dark for 1 hr. Finally, the samples were washed in PBT six times (5 min each). To double-stain proteins of interest, we incubated the samples in 0.1% hydrogen peroxide in PBT for 10 min to eliminate enzymatic activity in the TSA Plus Cyanine5 Evaluation kit, followed by standard immunostaining procedures (5).

Immunoblotting

For Western blot analysis, we homogenized exosome fractions in RIPA lysis buffer with protease inhibitor cocktails. Total protein (40 μg) was loaded in each lane of 4-12% Nupage Bis-Tris gels (Invitrogen) for immunoblotting. Mouse anti-TSG101 (1:1,000; Abcam), mouse anti-Syx1A (1:1,000; DSHB), mouse anti-Rab11 (1:1,000; BD Biosciences), horseradish peroxidase conjugated anti-mouse IgG (1:5,000; Jackson Lab) and Western Lightning Plus-ECL (PerkinElmer) were used for immunoblotting.

RT-PCR and real-time PCR

Total RNA was extracted from larvae or exosomal fractions using the miRNeasy Mini Kit (Qiagen) according to the manufacturer's instructions. SuperScript™ IV VILO™ Master Mix (Thermo Fisher Scientific) was used according to the manufacturer's instructions to detect mRNAs. To detect miR-274, a SuperScript™ III Reverse Transcriptase (Thermo Fisher Scientific) protocol was performed according to the manufacturer's instructions with the stem-loop RT primer that contains six nucleotides complementary to mature miR-274 3' sequences. For each reverse transcription reaction, 2 μg RNA was used. Phusion Green High-Fidelity DNA Polymerase (Thermo Fisher Scientific) was used to perform RT-PCR with specific primer sets for miR-274, *Ephrin* and *iav* (Figs. 3A, 3B, 3D and S5A). To measure *sty* mRNA levels (Fig 5C), we performed real-time PCR in 96-well plates using the LightCycler® 480 system. All mRNA transcripts were normalized to *Rpl19* transcript levels. To measure miR-274 levels (Figs. 3E, 3F and S5B), we conducted absolute qPCR by generating 8-point standard curves with miR-274 oligonucleotides in the range of 1 μM to 10^{-7} μM. Amounts of miR-274 were normalized to each respective *GAL4* control group and are shown as "fold change". All primers were listed in Table. S1.

RNA sequencing

Total RNA was extracted from wandering female larvae of w^{1118} and $mir\text{-}274^{KO}$ using a miRNeasy Mini Kit. Libraries were prepared with a TruSeq Stranded mRNA LT Sample Prep Kit for Illumina sequencing.

Luciferase construct and assay

Oligonucleotides for miR-274 targeting sequence of and mismatch control at the *sty* 3'-UTR were synthesized and inserted into the NheI/XhoI multiple cloning site of pmirGLO Dual-Luciferase miRNA target expression vector (Promega). Three plasmid constructs were generated: (1) without 3'-UTR sequences; (2) with wild-type 3'-UTR sequences; and (3) with mismatched 3'-UTR sequences. These plasmids (1 μg each) were transfected by the TransIT system (Mirus) into 2 x 10^6 S2 cells that were plated in 6-well plates one day before analysis. Dual-luciferase reporter assays (Promega) were performed 48 hr after transfection according to the manufacturer's instructions and measured by an EnSpire® Multimode Plate Reader.

Behavioral assay

Early- to mid-third instar larvae (AEL 72-96 hr) were used for the following behavioral

assays.

Larval crawling assay

Individual larvae were transferred to a 15 cm petri dish with 2% agarose for 1 min habituation. Activity was then video-recorded for 1 min using a Canon N90 camera and analyzed by Fiji with the wrMTrck plugin (9).

Feeding motivation assay

Larvae that were either well fed or food-deprived for 2 hr were transferred to a nutritious yeast or non-nutritious grape juice plate. Mouth-hook contractions of individual larvae were counted under dissecting microscopes for 1 min.

Hypoxia escape response

Hypoxia escape behavior was assessed as described (10), but with the following modifications. We used 10 larvae that had burrowed for 10 min into yeast paste applied to a grape juice agar plate. The assay was performed in a plexiglass chamber (20 x 10 x 10 cm) with O_2 levels of 21%, 10% or 1% regulated by N_2 infusion, which were monitored and controlled by a Proox 110 (BioSpherix) compact oxygen controller. Activity was video-recorded for 20 min using a Canon N90 camera and the number of larvae that moved out the yeast paste was counted at the 5-, 10-, and 15-min time points.

High-salt escape response

The experimental process was similar to hypoxia escape response with following modifications. Larvae that had burrowed for 10 min in yeast pastes that were further injected with 4M NaCl, and the percentages of fleeing larvae were assayed at 10 min.

Supplementary Figure legends

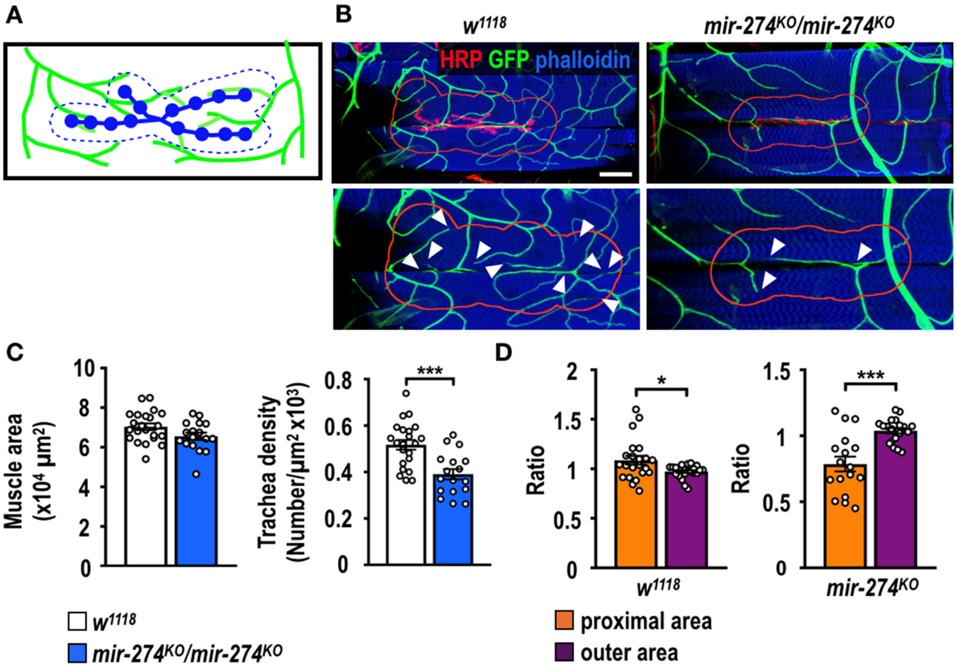

Fig. S1. Recruitment of tracheal branches to the proximal area of synaptic boutons. (A) Schematic depicts synaptic boutons (blue) and tracheal branches (green) at NMJ 6/7. Dotted curve line refers to the proximal area dilated from the outline of the synaptic boutons by 30 µm. (B) Confocal images show synaptic boutons (red), tracheal branches (green) and muscle (blue) at NMJ 6/7. The proximal areas were outlined in red curving lines in control w^{1118} and the *mir-274*^{KO} mutant. Arrow heads indicate tracheal terminal branches within the proximal area. Scale bar: 60 µm. (C) Bar graphs for quantification of muscle 6/7 areas in w^{1118} (70,391 ± 1,663 µm^2, n = 22) and *mir-274*^{KO}/*mir-274*^{KO} (65,558 ± 1,828 µm^2, n = 17), and for quantification of tracheal densities in w^{1118} (0.517 ± 0.021 branch/µm^2 x10^3, n = 22) and for *mir-274*^{KO}/*mir-274*^{KO} (0.391 ± 0.022 branch/µm^2 x10^3, n = 17). (D) Comparison of relative tracheal densities, which are normalized to respective overall tracheal densities, in the proximal and outer areas. w^{1118} (proximal, 1.09 ± 0.04; outer, 0.97 ± 0.01, n = 22) and the *mir-274*^{KO}/*mir-274*^{KO} (proximal, 0.786 ± 0.056; outer, 1.06 ± 0.0167, n = 17). (C and D) Data were analyzed by Independent *t*-tests. Statistical significances are shown as * for $p < 0.05$ and *** for $p < 0.001$.

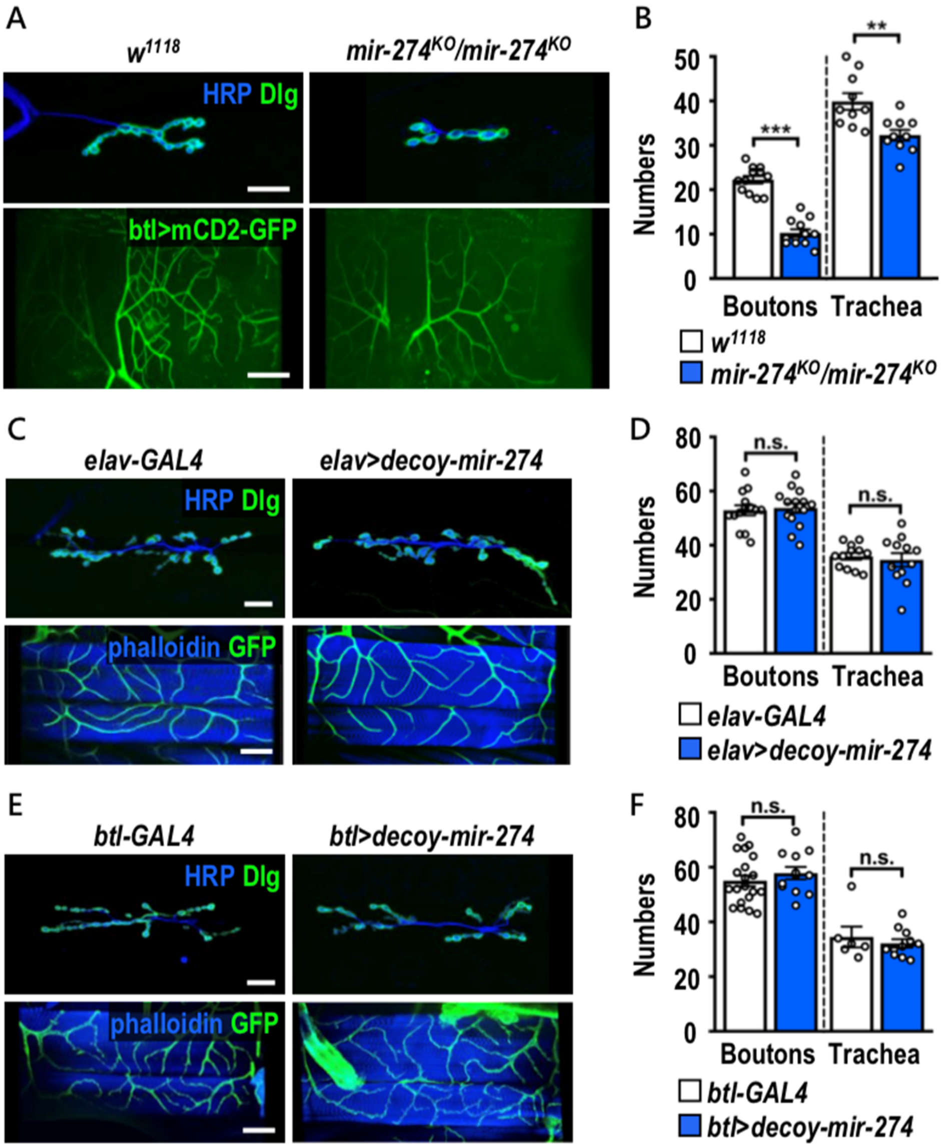

Fig. S2. Growth modulation of synaptic boutons and tracheal branches by miR-274. (A) Images of synaptic boutons immunostained for presynaptic HRP and postsynaptic Dlg at NMJs of muscle 4 (top panels, scale bar: 20 µm), and dorsal tracheal branches revealed by *btl-lexA>lexAop-CD2-GFP* (bottom panels, scale bar: 100 µm) in w^{1118} and $mir\text{-}274^{KO}/mir\text{-}274^{KO}$. (C and E) Images of synaptic boutons (scale bars: 30 µm) and tracheal branches (scale bars: 60 µm) for *elav-GAL4* (C) and *btl-GAL4* (E) controls (crossed to w^{1118}, left panels) or for miR-274 trapping (crossed to *UAS-decoy-mir-274,* right panels). (B, D and F) Bar graphs for quantification of synaptic boutons and tracheal branches. (B) Synaptic boutons for w^{1118}: 22.3 ± 0.85, n = 12, and for $mir\text{-}274^{KO}/mir\text{-}274^{KO}$: 10.3 ± 0.85, n = 12. Tracheal branches for w^{1118}: 39.9 ± 1.88, n = 10, and for $mir\text{-}274^{KO}/mir\text{-}274^{KO}$: 32.3 ± 1.24, n = 10. (D) Synaptic boutons for *elav-GAL4*: 52.9 ± 1.87, n = 14, and for *elav>decoy-mir-274*: 53.7 ± 1.78, n = 15. Tracheal branches for *elav-GAL4*: 35.9 ± 1.3, n = 12, and for *elav>decoy-mir-274*: 34.6 ± 2.51, n = 12.

(F) Synaptic boutons for *btl-GAL4*: 55 ± 1.95, n = 20, and for *btl>decoy-mir-274*: 57.8 ± 2.26, n =12. Tracheal branches for *btl-GAL4*: 34.5 ± 3.82, n = 6, and for *btl>decoy-mir-274*: 32.2 ± 1.54, n = 11. (B, D and F) Data were analyzed by Independent *t*-tests. Statistical significances are shown as n.s. for no significance, ** for $p < 0.01$ and *** for $p < 0.001$.

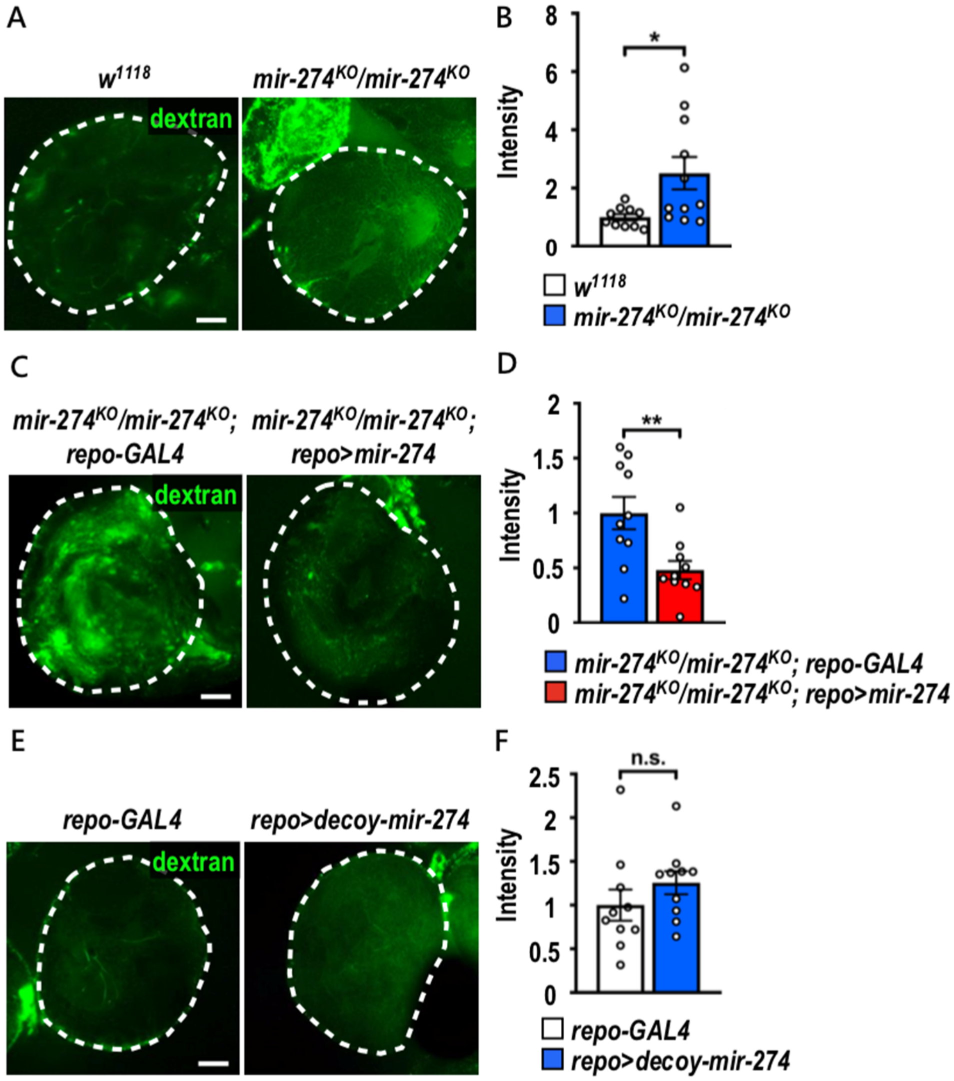

Fig. S3. Defective HBB restored by expressing miR-274 in glia. (A, C and E) The HBB integrity was assessed by immersing dissected larval fillets with labeled dextran (Dextran, Alexa Fluor™ 647; 10,000 MW, Thermo Fisher Scientific) for 30 min, and the leakage of the dextran dye into the larval brain (dotted line) was imaged (11). In pairwise comparisons, higher levels of fluorescence intensity were detected in *mir-274*^{KO}/*mir-274*^{KO} compared to *w*^{1118} (A), and in *mir-274*^{KO}/*mir-274*^{KO}; *repo-GAL4*, compared to glial-rescue *mir-274*^{KO}/*mir-274*^{KO}; *repo>mir-274* (C). However, *repo-GAL4* and *repo>decoy-mir-274* show comparable intensities (E). Scale bars: 30 µm. (B, D and F) Bar graphs for quantification of relative fluorescence intensities with controls set to 1. (B) *w*^{1118}: 1 ± 0.109, n = 10, *mir-274*^{KO}/*mir-274*^{KO}: 2.51 ± 0.557, n =11. (D) *mir-274*^{KO}/*mir-274*^{KO}*; repo-GAL4:* 1 ± 0.148, n = 10, and *mir-274*^{KO}/*mir-274*^{KO}*; repo>mir-274*: 0.48 ± 0.08, n =10. (F) *repo-GAL4*: 1 ± 0.178, n = 10, and *repo>decoy-mir-274*: 1.26 ± 0.133, n = 10. Data was analyzed by Independent *t*-tests. Statistical significances are shown as n.s. for no significance, * for $p < 0.05$ and ** for $p < 0.01$.

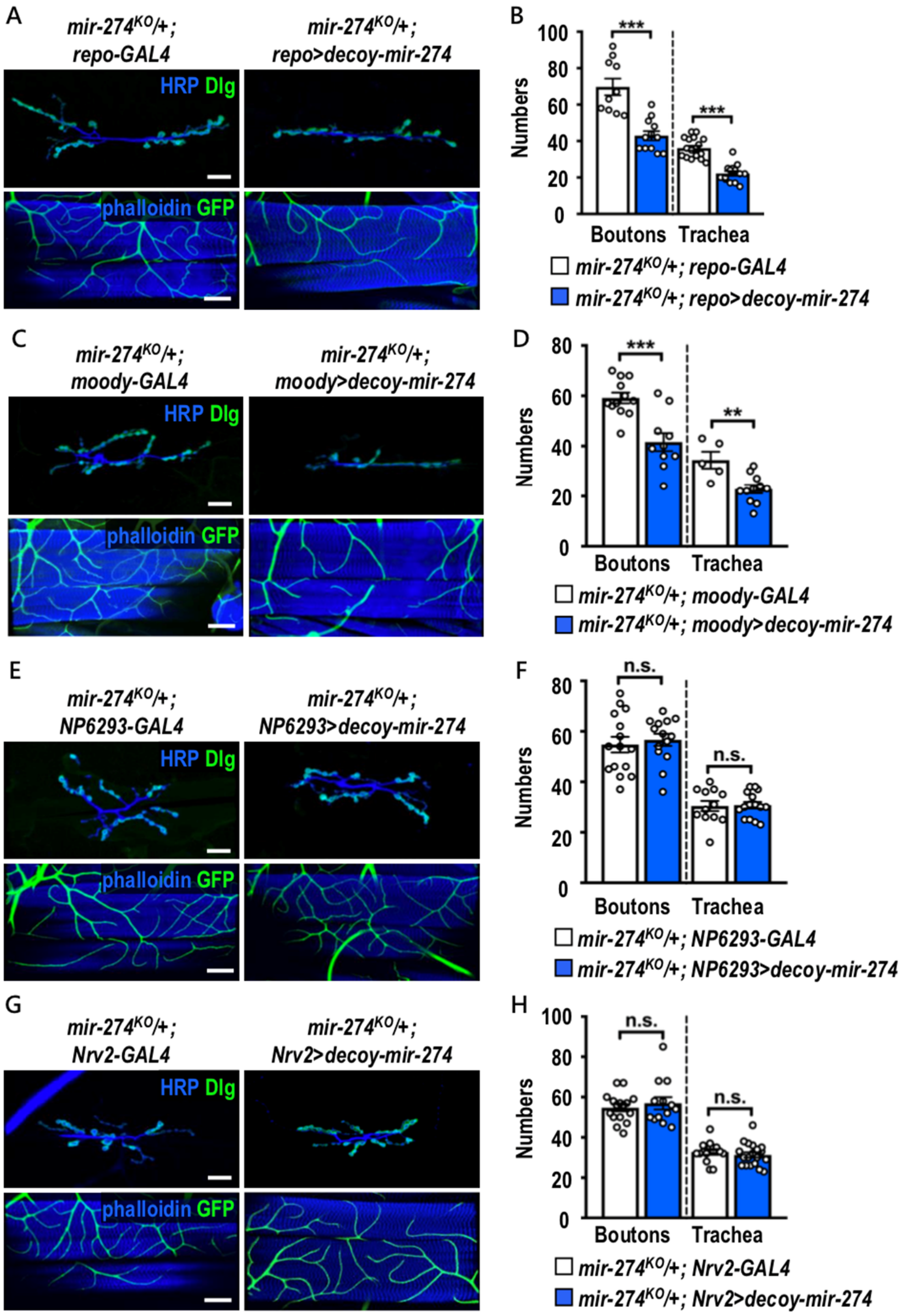

Fig. S4. Subperineurial glia in modulating synaptic and tracheal growth. (A, C, E and G) Confocal images of synaptic boutons (scale bars: 30 µm) and tracheal branches (scale bars: 60 µm) in *mir-274*^{KO}*/+; repo-GAL4* and *mir-274*^{KO}*/+; repo>decoy-mir-274* (A), *mir-274*^{KO}*/+; moody-GAL4* and *mir-274*^{KO}*/+; moody>decoy-mir-274* (C), *mir-274*^{KO}*/+; NP6293-GAL4* and *mir-274*^{KO}*/+; NP6293>decoy-mir-274* (E), and *mir-274*^{KO}*/+; Nrv2-GAL4* and *mir-274*^{KO}*/+; Nrv2>decoy-mir-274* (G). (B, D, F and H) Bar graphs for quantifications of synaptic boutons and tracheal branches. (B) Synaptic boutons for *mir-274*^{KO}*/+; repo-GAL4*: 69.7 ± 4.61, n = 10, and *mir-274*^{KO}*/+; repo>decoy-mir-274*: 42.9 ± 2.54, n = 12. Tracheal branches for *mir-*

274^{KO}*/+; repo-GAL4*: 36.1 ± 1.36, n = 17, and *mir-274*^{KO}*/+; repo>decoy-mir-274*: 22.2 ± 1.31, n = 14. (D) Synaptic boutons for *mir-274*^{KO}*/+; moody-GAL4*: 59.2 ± 2.1, n = 12, and *mir-274*^{KO}*/+; moody>decoy-mir-274*: 41.6 ± 3.57, n = 10. Tracheal branches for *mir-274*^{KO}*/+; moody-GAL4*: 34.4 ± 3.37, n = 5, and *mir-274*^{KO}*/+; moody>decoy-mir-274*: 22.8 ± 1.68, n = 11. (F) Synaptic boutons for *mir-274*^{KO}*/+; NP6293-GAL4*: 54.7 ± 3.07, n = 15, and *mir-274*^{KO}*/+; NP6293>decoy-mir-274*: 56.6 ± 2.41, n = 14. Tracheal branches for *mir-274*^{KO}*/+; NP6293-GAL4*: 30.4 ± 1.99, n = 12, and *mir-274*^{KO}*/+; NP6293>decoy-mir-274*: 30.7 ± 1.31, n = 15. (H) Synaptic boutons for *mir-274*^{KO}*/+; Nrv2-GAL4*: 54.7 ± 1.76, n = 16, and *mir-274*^{KO}*/+; Nrv2>decoy-mir-274*: 56.9 ± 3.07, n = 13. Tracheal branches for *mir-274*^{KO}*/+; Nrv2-GAL4*: 32.8 ± 1.38, n = 14, and *mir-274*^{KO}*/+; Nrv2>decoy-mir-274*: 31.3 ± 1.3, n = 19. Data were analyzed by Independent *t*-tests. Statistical significances are shown as n.s. for no significance, ** for $p < 0.01$ and *** for $p < 0.001$.

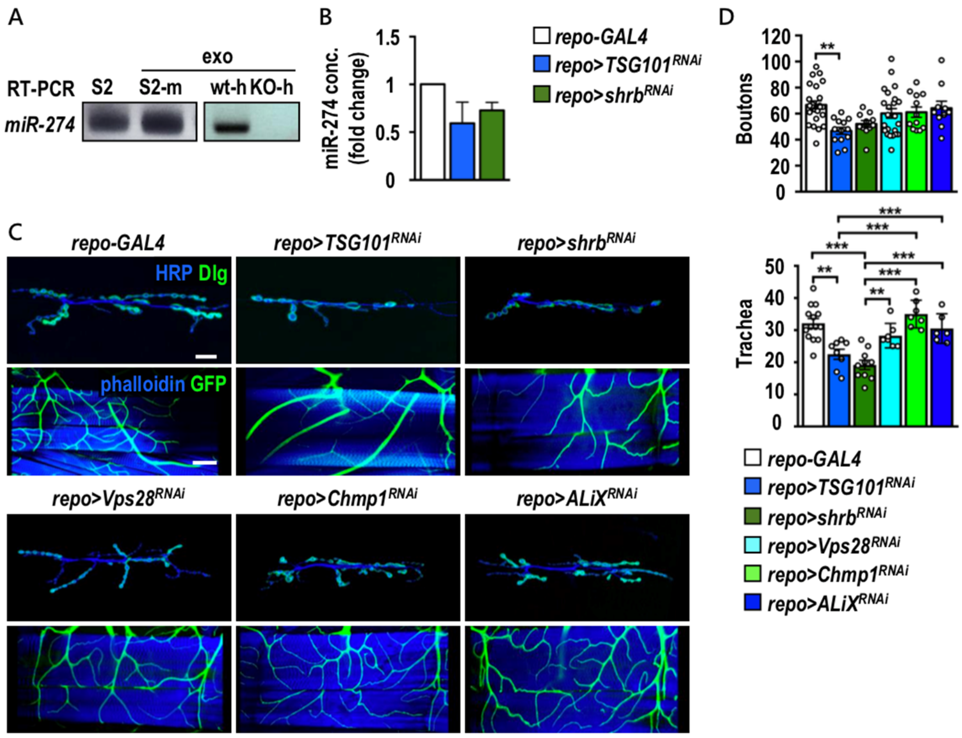

Fig. S5. Components of ESCRT complexes in glial secretion of exosomal miR-274. (A) miR-274 was detected by RT-PCR in total S2 cell lysates (S2) as control, and in exosomal fractions isolated from medium used to culture S2 cells (S2-m) and in wild-type larval hemolymph (wt-h). However, no signal was detected in exosomal fractions from *mir-274*^{KO} hemolymph (KO-h). (B) Bar graph shows quantification of absolute qPCR to detect the miR-274 levels in exosomal fractions prepared from larval hemolymphs. The experiments were repeated (n = 3), and the number of *repo-GAL4* in each experiment was used for normalization to *repo>TSG101*^{RNAi} (0.593 ± 0.221) and *repo>shrb*^{RNiA} (0.728 ± 0.084). (C) Confocal images of synaptic boutons (scale bar: 30 μm) and tracheal branches (scale bar: 60 μm) in *repo-GAL4*, *repo>TSG101*^{RNAi}, *repo>shrb*^{RNiA}, *repo>Vps28*^{RNAi}, *repo>Chmp1*^{RNAi} and *repo>ALiX*^{RNAi}. (D) Bar graphs for quantifications of synaptic boutons and tracheal branches. Synaptic boutons for *repo-GAL4*: 66.7 ± 3.05 (n = 24), *repo>TSG101*^{RNAi}: 46.8 ± 2.66 (n = 13), *repo>shrb*^{RNAi}: 52 ± 2.63 (n = 11), *repo>Vps28*^{RNAi}: 60 ± 3.71 (n = 23), *repo>Chmp1*^{RNAi}: 61.1 ± 3.94 (n = 12) and *repo>ALiX*^{RNAi}: 64.2 ± 5.32 (n = 10). Tracheal branches for *repo-GAL4*: 32.1 ± 1.46 (n = 14), *repo>TSG101*^{RNAi}: 22.5 ± 1.58 (n = 8), *repo>shrb*^{RNAi}: 19.2 ± 1.47 (n = 10), *repo>Vps28*^{RNAi}: 28.3 ± 1.44 (n = 7), *repo>Chmp1*^{RNAi}: 35 ± 1.63 (n = 7) and *repo>ALiX*^{RNAi}: 30.5 ± 1.88 (n = 6). Color coding for bars is shown below. Data were analyzed by One-way ANOVA followed by Tukey post hoc. Statistical significances are shown as ** for $p < 0.01$ and *** for $p < 0.001$.

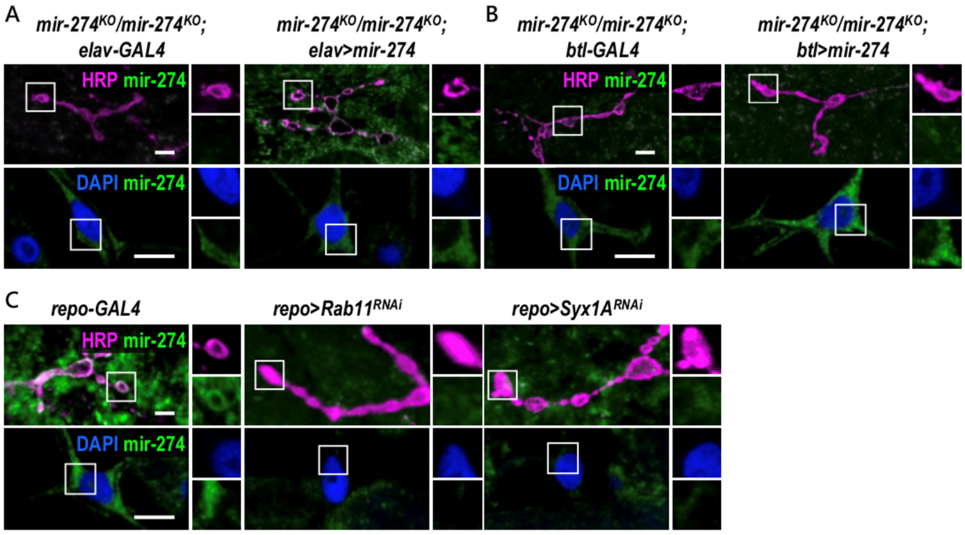

Fig. S6. Restricted distributions of miR-274. (A-C) Confocal images show miR-274 FISH signals in synaptic boutons (top panels, scale bars: 5 μm) and tracheal cells (bottom panels, scale bars: 10 μm). Boxed areas are enlarged in right panels. (A and B) Neuronal *elav-GAL4* (A) or tracheal *btl-GAL4* (B) in the *mir-274*^{KO}*/mir-274*^{KO} mutants shows background signals (left panels). (A) When driving the *UAS-mir-274* transgene by *elav-GAL4*, FISH signals appear in synaptic boutons and muscles but not in tracheal cells. (B) When driving *UAS-mir-274* by *btl-GAL4*, FISH signals appear only in tracheal cells. (C) FISH signals appear in synaptic boutons and tracheal cells in *repo-GAL4* control, which are largely diminished in glial knockdown of Rab11 (*repo>Rab11*^{RNAi}) or Syx1A (*repo>Syx1A*^{RNAi}).

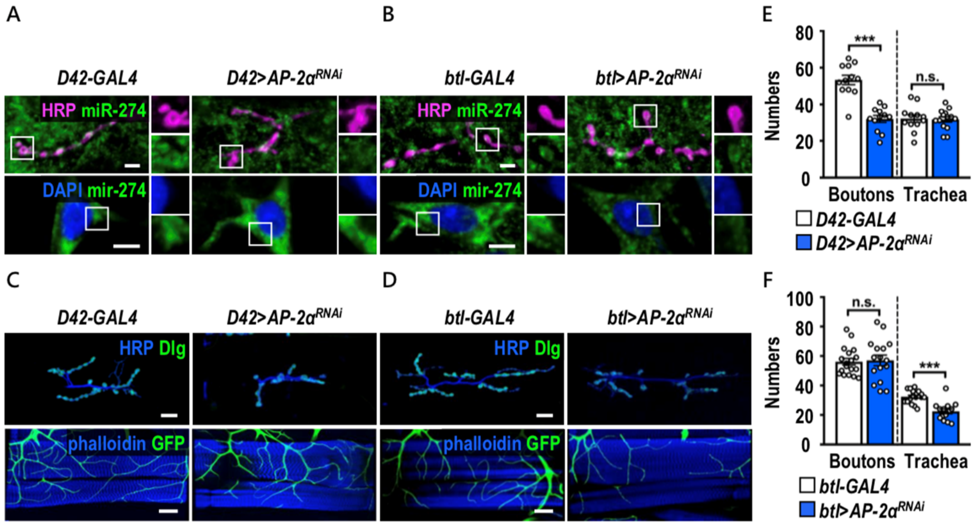

Fig. S7. miR-274 localization and function in AP-2α knockdown in target cells. (A and B) Confocal images show miR-274 FISH signals in synaptic boutons (top panels, scale bar: 5 µm) and tracheal cells (bottom panels, scale bar: 10 µm) when endocytosis was blocked in motor neurons (A) and tracheal cells (B). (A) The FISH signals were specifically reduced in motor neurons in *D42>AP-2α^RNAi^* knockdown as compared to the driver *D42-GAL4* control. (B) The FISH signals were specifically reduced in tracheal cells in *btl>AP-2α^RNAi^* knockdown as compared to the driver *btl-GAL4* control. Boxed areas are enlarged in the right. (C and D) Images show synaptic boutons (top panels, scale bar: 30 µm) and tracheal branches (bottom panels, scale bar: 60 µm) in *D42-GAL4* and *D42>AP-2α^RNAi^* (C), and *btl-GAL4* and *btl>AP-2α^RNAi^* (D). (E and F) Bar graphs show quantifications for synaptic boutons and tracheal branches for *D42-GAL4* (E) and *btl-GAL4* (F) knockdown. (E) Synaptic boutons for *D42-GAL4*: 53.3 ± 2.56 (n = 12) and *D42>AP-2α^RNAi^*: 31.9 ± 1.9 (n = 12). Tracheal branches for *D42-GAL4*: 32.1 ± 2.02 (n = 12) and *D42>AP-2α^RNAi^*: 31.6 ± 1.46 (n = 14). (F) Synaptic boutons for *btl-GAL4*: 56.1 ± 2.25 (n = 18) and *btl>AP-2α^RNAi^*: 57.1 ± 3.58 (n = 16). Tracheal branches for *btl-GAL4*: 32.4 ± 0.997 (n = 18) and *btl>AP-2α^RNAi^*: 22.4 ± 1.66 (n = 14). Data was analyzed by Independent *t*-tests. Statistical significance is shown as n.s. for no significance and *** for $p < 0.001$.

A

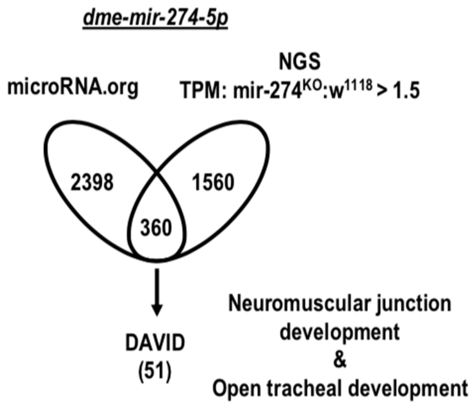

B

FlybaseID	Name	Symbol
FBgn0029094	anti-silencing factor 1	asf1
FBgn0015903	apontic	apt
FBgn0000099	apterous	ap
FBgn0027620	ATP-dependent chromatin assembly factor large subunit	Acf
FBgn0004624	Calcium/calmodulin-dependent protein kinase II	CaMKII
FBgn0010434	coracle	cora
FBgn0016131	Cyclin-dependent kinase 4	Cdk4
FBgn0010316	dacapo	dap
FBgn0027538	β1,4-N-acetylgalactosaminyltransferase A	β4GalNAcTA
FBgn0000546	Ecdysone receptor	EcR
FBgn0000547	echinoid	ed
FBgn0005427	erect wing	ewg
FBgn0000575	extra macrochaetae	etc
FBgn0000635	Fasciclin 2	FasII
FBgn0024236	fear-of-intimacy	foi
FBgn0053556	formin 3	form3
FBgn0000658	four-jointed	fj
FBgn0004652	fruitless	fru
FBgn0004919	goliath	gol
FBgn0259211	grainy head	grh
FBgn0017397	held out wings	how
FBgn0004873	hu li tai shao	hts
FBgn0267002	uncoordinated-104	unc-104
FBgn0001311	krotzkopf verkehrt	kkv
FBgn0011648	Mothers against dpp	Mad
FBgn0004456	multiple edematous wings	mew

FlybaseID	Name	Symbol
FBgn0002789	Muscle protein 20	Mp20
FBgn0053197	muscleblind	mbl
FBgn0015513	myoblast city	mbc
FBgn0002921	Na pump alpha subunit	Atpα
FBgn0005636	nervy	nvy
FBgn0013997	Neurexin IV	Nrx-IV
FBgn0014001	p21-activated kinase	Pak
FBgn0085432	pangolin	pan
FBgn0003118	pointed	pnt
FBgn0243512	puckered	puc
FBgn0010379	Akt1	Akt1
FBgn0003396	schnurri	shn
FBgn0003507	serpent	srp
FBgn0003334	Sex comb on midleg	Scm
FBgn0003392	shibere	shi
FBgn0027363	Signal transducing adaptor molecule	Stam
FBgn0010894	sinuous	sinu
FBgn0014388	sprouty	sty
FBgn0086897	squid	sqd
FBgn0004603	Src oncogene at 42A	Src42A
FBgn0003525	string	stg
FBgn0015838	Van Gogh	Vang
FBgn0086680	ventral veins lacking	vvl
FBgn0024179	wishful thinking	wit
FBgn0010453	Wnt oncogene analog 4	Wnt4

Fig. S8. *sty* as a target gene for miR-274. (A) Experimental approaches for miR-274 target gene search and selection. In one approach, potential miR-274 target genes were predicted and annotated by miRanda (www.microRNA.org). In another approach, genes upregulated in homozygous *mir-274*^{KO} larvae by NGS (> 1.5 folds relative to w^{1118}) were selected. Overlapping candidates were further filtered by DAVID Bioinformatics Resources 6.7 (david-

d.ncifcrf.gov) for functions in NMJ and tracheal development. (B) List of 51 miR-274 possible targets.

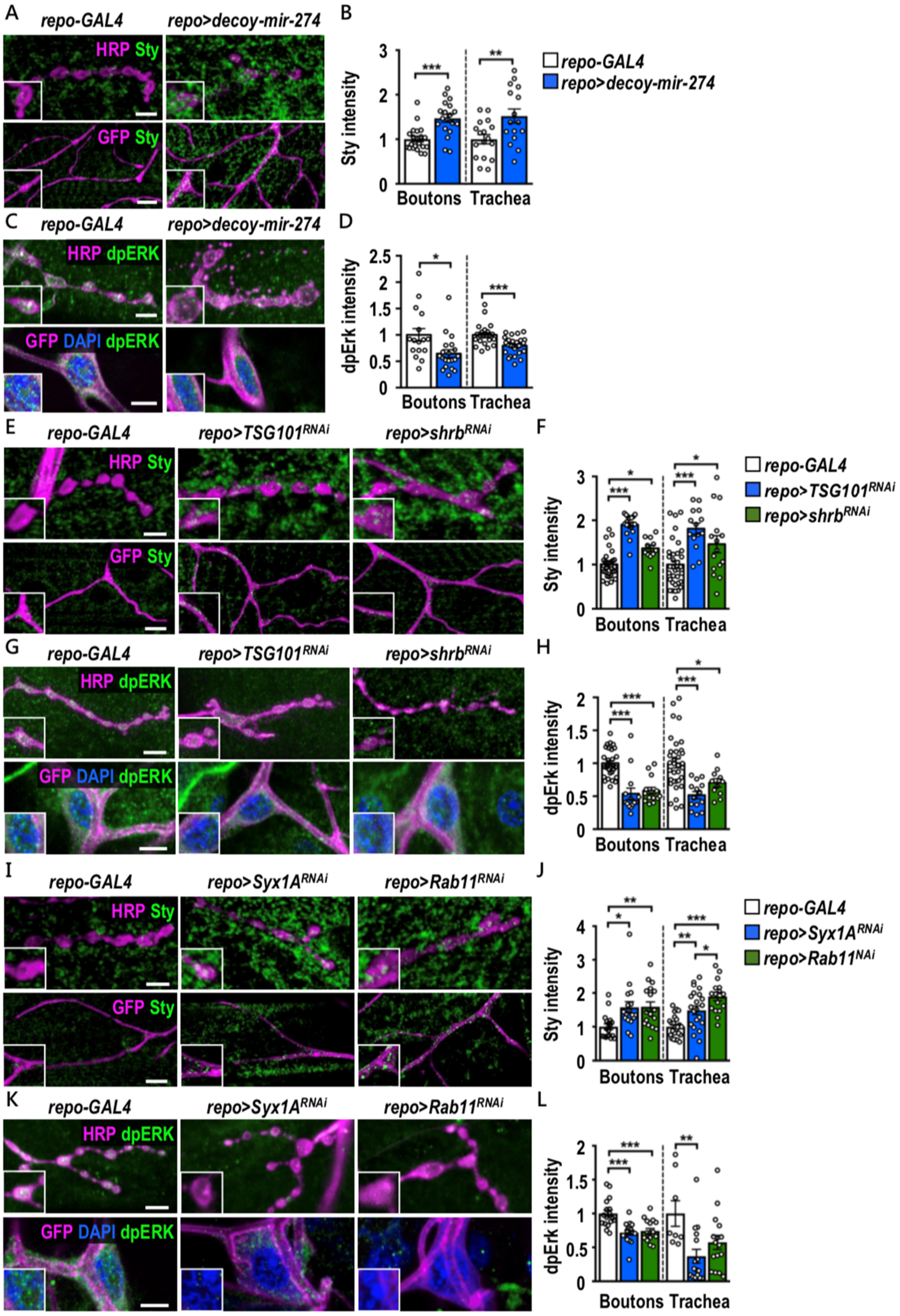

Fig. S9. Glial ESCRT components, Syx1A and Rab11 modulate Sty and dpERK levels in synaptic boutons and tracheal cells. (A, C, E, G, I and K) Confocal images showing immunostaining for Sty (A, E and I) or dpERK (C, G and K) in synaptic boutons and tracheal branches in *repo-GAL4* and *repo>decoy-mir-274* (A and C), *repo-GAL4*, *repo>TSG101*^{RNAi} and *repo>shrb*^{RNAi} (E and G), and *repo-GAL4*, *repo>Syx1A*^{RNAi} and *repo>Rab11*^{RNAi} (I and K). (B, D, F, H, J and L) Bar graphs for quantifications for Sty (B, F and J) and dpERK (D, H, and L) levels in synaptic boutons and tracheal cells, and normalized to respective controls. (B) *repo-GAL4* (synaptic boutons: 1 ± 0.06, n = 21; tracheal cells: 1 ± 0.10, n = 17) and

repo>decoy-mir-274 (synaptic boutons: 1.47 ± 0.088, n = 19; tracheal cells: 1.52 ± 0.156, n = 16). (D) *repo-GAL4* (synaptic boutons: 1 ± 0.12, n = 16; tracheal cells: 1 ± 0.04, n = 22) and *repo>decoy-mir-274* (synaptic boutons: 0.65 ± 0.074, n = 20; tracheal cells: 0.796 ± 0.032, n = 27). (F) *repo-GAL4* (synaptic boutons: 1 ± 0.058, n = 29; tracheal cells: 1 ± 0.089, n = 34), *repo>TSG101*^{RNAi} (synaptic boutons: 1.9 ± 0.053, n = 19; tracheal cells: 1.81±0.125, n=14) and *repo>shrb*^{RNAi} (synaptic boutons: 1.37 ± 0.072, n = 11; tracheal cells: 1.46 ± 0.19, n = 15). (H) *repo-GAL4* (synaptic boutons: 1 ± 0.041, n = 28; tracheal cells: 1 ± 0.071, n = 34), *repo>TSG101*^{RNAi} (synaptic boutons: 0.537 ± 0.086, n = 14; tracheal cells: 0.516 ± 0.056, n = 14) and *repo>shrb*^{RNAi} (synaptic boutons: 0.583 ± 0.0518, n = 14; tracheal cells: 0.703 ± 0.0631, n = 11). (J) *repo-GAL4* (synaptic boutons: 1 ± 0.086, n = 18; tracheal cells: 1 ± 0.0641, n = 24), *repo>Syx1A*^{RNAi} (synaptic boutons: 1.57 ± 0.167, n = 17; tracheal cells: 1.49 ± 0.132, n = 22) and *repo>Rab11*^{RNAi} (synaptic boutons: 1.59 ± 0.144, n = 18; tracheal cells: 1.9 ± 0.123, n = 16). (L) *repo-GAL4* (synaptic boutons: 1 ± 0.051, n = 17; tracheal cells: 1 ± 0.19, n = 8), *repo>Syx1A*^{RNAi} (synaptic boutons: 0.72 ± 0.038, n = 16; tracheal cells: 0.37 ± 0.102, n = 16) and *repo>Rab11*^{RNAi} (synaptic boutons: 0.742 ± 0.378, n = 17; tracheal cells: 0.574 ± 0.104, n = 16). Data was analyzed by Independent *t*-tests (B and D) and One-way ANOVA followed by Tukey post hoc (F, H, J and L). Statistical significances are shown as * for $p < 0.05$, ** for $p < 0.01$ and *** for $p < 0.001$.

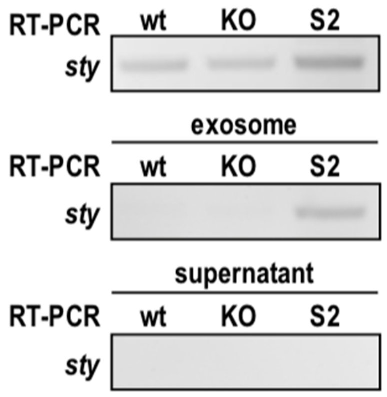

Fig. S10. Absence of *sty* mRNA in exosomes isolated from larval hemolymph. RT-PCR for measuring *sty* mRNA expressions in total lysates, fractionated exosomes, and exosome-depleted supernatants prepared from wild-type w^{1118} (wt), homozygous $mir\text{-}274^{KO}$ (KO) larvae and S2 cells (S2).

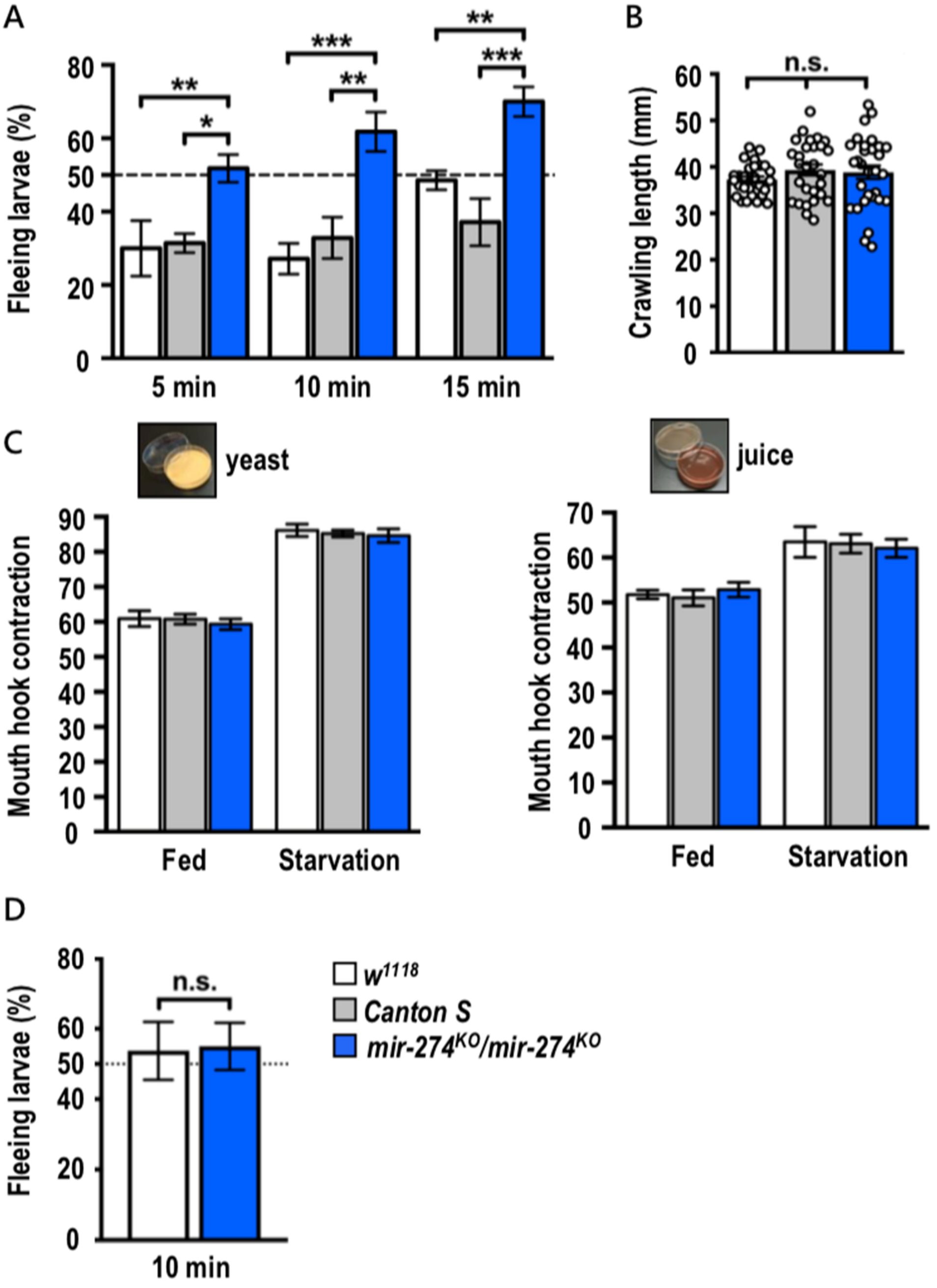

Fig. S11. Responses of miR-274 mutant larvae to different cues. (A) Bar graph shows higher percentages of fleeing *mir-274*^{KO} larvae as compared to controls *w*^{1118}, *Canton S* at 5, 10 and 15 minutes in response to 1% O_2 with 10% nutrition. Percentages at 5 min for *w*^{1118} (30 ± 7.56, n = 7), *Canton S* (31.4 ± 2.61, n = 7) and *mir-274*^{KO}*/mir-274*^{KO} (51.8 ± 3.77, n = 11), at 10 min for *w*^{1118} (27.1 ± 4.21, n = 7), *Canton S* (32.9 ± 5.65, n = 7) and *mir-274*^{KO}*/mir-274*^{KO} (61.8 ± 5.36, n = 11), and at 15 min for *w*^{1118} (48.6 ± 2.61, n = 7), *Canton S* (37.1 ± 6.44, n = 7) and *mir-274*^{KO}*/mir-274*^{KO} (70 ± 4.05, n = 11). (B) Bar graph shows comparable crawling lengths in 1 minute among *w*^{1118} (37.3 ± 0.668, n = 27), *Canton S* (39.3 ± 1.18, n = 28) and *mir-274*^{KO}*/mir-274*^{KO} (38.8 ± 1.42, n = 30) larvae. (C) Bar graphs show no significant differences

in the frequencies of mouth hook contractions in fed or starved larvae in yeast or grape juice plates. Contractions in 1 minute in yeast plates for *w*^{1118} (fed: 60.95 ± 2.25, n = 20; starved: 86.17 ± 1.764, n = 18), *Canton S* (fed: 59.67 ± 1.448, n = 21; starved: 85.26 ± 0.9729, n = 19) and *mir-274*^{KO}/*mir-274*^{KO} (fed: 58.57 ± 1.394, n = 23; starved: 84.64 ± 1.942, n = 22), and in grape juice plates for *w*^{1118} (fed: 51.8 ± 0.9832, n = 25; starved: 63.48 ± 3.427, n = 27), *Canton S* (fed: 51.05 ± 1.773, n = 20; starved: 63.1 ± 2.111, n = 20) and *mir-274*^{KO}/*mir-274*^{KO} (fed: 52.85 ± 1.679, n = 27; starved: 62.07 ± 2.019, n = 27). (D) Bar graph shows no significant differences of fleeing larvae at 10 minutes in the high salt condition. Percentages for *w*^{1118} (53.8 ± 8.22, n = 8) and *Canton S* (55 ± 6.69, n = 12). Data were analyzed by One-way ANOVA followed by Tukey post hoc tests (A and B), Two-way ANOVA followed by Tukey post hoc tests (C), or Independent *t*-tests (D). Statistical significances are shown as n.s. for no significance, * for $p < 0.05$, ** for $p < 0.01$ and *** for $p < 0.001$.

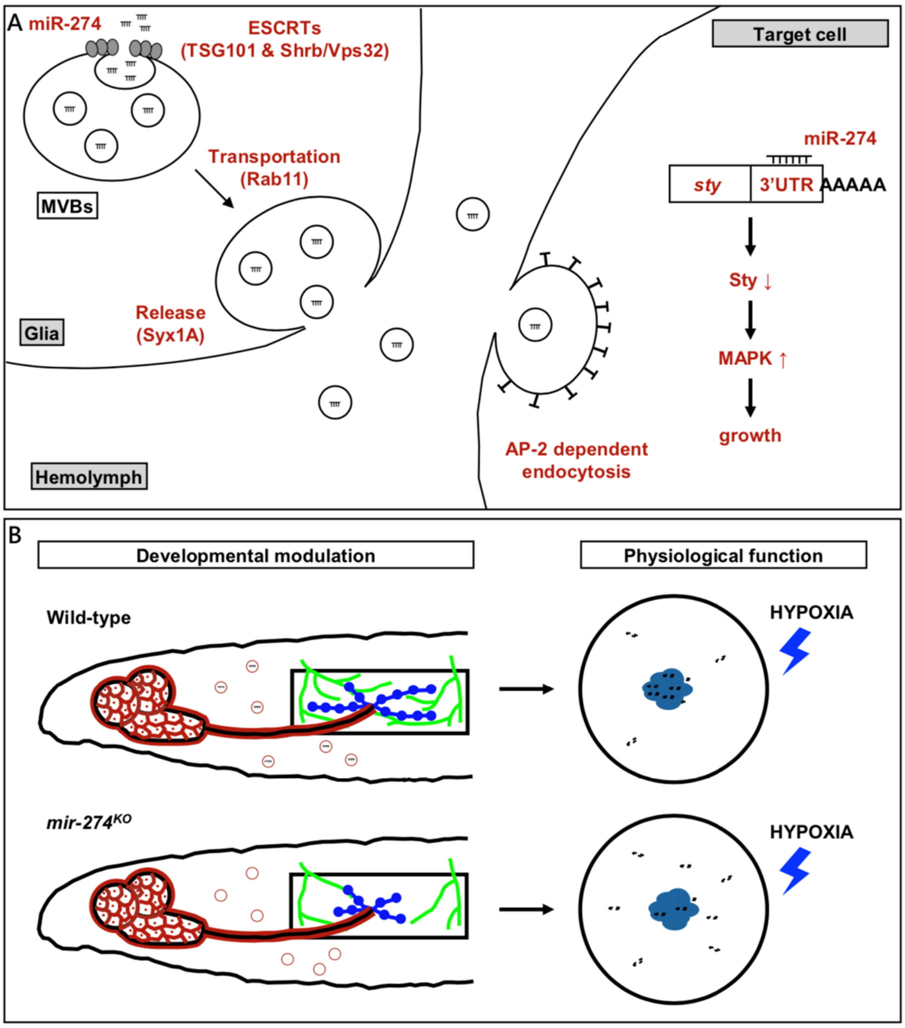

Figure S12. Model for exosomal miR-274 in secretion, circulation and targeting in growth and behavior modulation. (A) Diagram depicting the sorting and packaging of miR-274 into intraluminal vesicles within MVBs through ESCRT-dependent mechanisms. The mature exosomes within MVBs are transported and released extracellularly through Rab11- and Syx1A-dependent mechanisms. Secreted miR-274-containing exosomes in the circulating hemolymph are uptaken into neurons and tracheal cells through AP-2α dependent endocytosis machinery, where miR-274 targets *sty* 3'-UTR to downregulate Sty expression. Sty downregulation within target cells results in upregulation of MAPK signaling, thus promoting growth of synaptic boutons and tracheal branches. (B) During larval development, glia (red) secret miR-274-carrying exosomes (red circles) to promote growth of synaptic boutons (blue) and tracheal branches (green). The intact tracheal system is required for larvae to tolerate low oxygen conditions (hypoxia) and stay in the food paste (dark blue). Lacking miR-274 in the *miR-274*^{KO} mutant causes reduced tracheal branches (and synaptic boutons) that leads to intolerance to hypoxia and fleeing away from the food source.

Supplementary Table

Table. S1. List of oligonucleotides.

miR-274 oligonucleotides	GTC GTA TCC AGT GCA GGG TCC GAG GTA TTC GCA CTG GAT ACG ACT ACC CGT TAG TGT CGG TCA CAA ACG CGT G
Stem-loop RT primer	GTC GTA TCC AGT GCA GGG TCC GAG GTA TTC GCA CTG GAT ACG ACT ACC CG
miR-274 forward	CAC GCG TTT GTG ACC GAC ACT AAC
miR-274 Stem-loop reverse	CCA GTG CAG GGT CCG AGG TA
Ephrin forward	TCC CAT AAG GGT AAT GCT AAC AG
Ephrin reverse	ACC TCG TTG GGA TGT TTG TT
iav forward	GTT GTC CGC CTT TCT CAA G
iav reverse	CAG CAA GTT GGA GAA CAG GAA
sty forward	ACC ACG GAT TGC TTT TGA A
sty reverse	AGT TAC TGC GCC CGG ACT
rpl19 forward	TCT CTA AAG CTC CAG AAG AGG C
rpl19 reverse	CGA TCT CGT TGA TTT CAT TGG GA
miR-274 precursor probe	ATT GTT TGG CCG CCA GGA TTA
sty luciferase forward	CTA GCA ACT GAA AAC TGA AAA TTA CAC AAA AC
sty luciferase reverse	TCG AGT TTT GTG TAA TTT TCA GTT TTC AGT TG
mismatch sty luciferase forward	CTA GCA ACT GAA AAC TGA AAA TTA TTT TTT TC
mismatch sty luciferase reverse	TCG AGA AAA AAA TAA TTT TCA GTT TTC AGT TG
Decoy-linker-mir-274 forward	TCT GAA TAG GGA ATT CCG CCG CTA GCT TTA AAG TCC ACA ACT CAT CAA GGA AAA TGG ACG GCG CTA GGA TCA TCA AC

Decoy-linker-mir-274 reverse	CCT CGA GCC GCG GCC GCA CAC TAG TGA CGG CGC TAG GAT CAT CTT G
Decoy-function-mir-274 forward	GAC GGC GCT AGG ATC ATC AAC ATT ACC CGT TAG TGT CAG CAG GTC ACA AAA CAA GTA TTC TGG TCA CAG AAT ACA ACA TTA
Decoy-function-mir-274 reverse	GAC GGC GCT AGG ATC ATC TTG TTT TGT GAC CTG CTG ACA CTA ACG GGT AAT GTT GTA TTC TGT GAC CAG AAT ACT TGT TTT

Table S2. Summary of statistical analyses.

Figure 1

**1F (Boutons); left panel**		
**Genotypes**	**n**	**Mean ± SEM**
w^{1118}	22	62.3 ± 2.39
mir-274^{KO}/+	16	59.5 ± 2.43
mir-274^{KO}/mir-274^{KO}	15	36.4 ± 2.31
mir-274^{KO}/mir-274$^{6-3}$	15	46.9 ± 2.28
**1F (Trachea); right panel**		
**Genotypes**	**n**	**Mean ± SEM**
w^{1118}	16	34.94 ± 2.071
mir-274^{KO}/+	11	31.55 ± 1.557
mir-274^{KO}/mir-274^{KO}	13	20.46 ± 1.859
mir-274^{KO}/mir-274$^{6-3}$	11	23.09 ± 1.498

**1G (Boutons); left panel**		
**Genotypes**	**n**	**Mean ± SEM**
repo-GAL4	18	63.8 ± 2.49
repo>decoy-mir-274	20	49 ± 1.41
**1G (Trachea); right panel**		
**Genotypes**	**n**	**Mean ± SEM**
repo-GAL4	11	32.4 ± 1.74
repo>decoy-mir-274	20	23.3 ± 1.37

**1H (Boutons); left panel**		
**Genotypes**	**n**	**Mean ± SEM**
mir-274^{KO}/+; repo-GAL4	8	54.5 ± 3.65
mir-274^{KO}/+; UAS-mir-274	11	57.4 ± 2.09
mir-274^{KO}/mir-274^{KO}; repo-GAL4	14	42.1 ± 2.09
mir-274^{KO}/ mir-274^{KO}; UAS-mir-274	9	45.9 ± 2.09
mir-274^{KO}/mir-274^{KO}; repo>mir-274	9	55.1 ± 2.78
**1H (Trachea); right panel**		

Genotypes	n	Mean ± SEM
mir-274^{KO}*/+; repo-GAL4*	12	33.8 ± 2.05
mir-274^{KO}*/+; UAS-mir-274*	13	33.8 ± 1.91
mir-274^{KO}*/mir-274*^{KO}*; repo-GAL4*	14	20.1 ± 1.74
mir-274^{KO}*/ mir-274*^{KO}*; UAS-mir-274*	12	18.8 ± 1.89
mir-274^{KO}*/mir-274*^{KO}*; repo>mir-274*	16	29.6 ± 0.913

Figure 3

**3E**		
**Genotypes**	**n**	**Mean ± SEM**
mir-274^{KO}*/mir-274*^{KO}*; repo>mir-274*	2	3.04 ± 1.55
mir-274^{KO}*/mir-274*^{KO}*; elav>mir-274*	2	1.43 ± 0.7
mir-274^{KO}*/mir-274*^{KO}*; btl>mir-274*	2	1.6 ± 0.265

**3F**		
**Genotypes**	**n**	**Mean ± SEM**
repo-GAL4	3	1
repo>Syx1A^{RNAi}	3	0.0601 ± 0.031
repo> Rab11^{RNAi}	3	0.08 ± 0.0214

**3H (Boutons); left panel**		
**Genotypes**	**n**	**Mean ± SEM**
repo-GAL4	16	62.3 ± 3.72
repo>Syx1A^{RNAi}	12	41.6 ± 2.74
repo>Rab11^{RNAi}	16	37.4 ± 2.79
**3H (Trachea); right panel**		
**Genotypes**	**n**	**Mean ± SEM**
repo-GAL4	12	39.8 ± 1.58
repo>Syx1A^{RNAi}	11	21 ± 2.17
repo>Rab11^{RNAi}	14	23.9 ± 1.79

Figure 4

**4E (Boutons); left panel**

Genotypes	n	Mean ± SEM
mir-274^{KO}*/+; elav-GAL4*	27	67.74 ± 2.389
mir-274^{KO}*/+; elav>decoy-mir-274*	12	49.25 ± 4.535
**4E (Trachea); right panel**		
**Genotypes**	**n**	**Mean ± SEM**
mir-274^{KO}*/+; elav-GAL4*	29	35.1 ± 1.3
mir-274^{KO}*/+; elav>decoy-mir-274*	19	36.8 ± 1.05

**4F (Boutons); left panel**		
**Genotypes**	**n**	**Mean ± SEM**
mir-274^{KO}*/+; btl-GAL4*	28	61.9 ± 2.61
mir-274^{KO}*/+; btl>decoy-mir-274*	11	65.3 ± 2.37
**4F (Trachea); right panel**		
**Genotypes**	**n**	**Mean ± SEM**
mir-274^{KO}*/+; btl-GAL4*	31	32.6 ± 1.26
mir-274^{KO}*/+; btl>decoy-mir-274*	15	21.9 ± 1.51

**4H (Boutons); left panel**		
**Genotypes**	**n**	**Mean ± SEM**
mir-274^{KO}*/ mir-274*^{KO}*; elav-GAL4*	16	44.6 ± 2.56
mir-274^{KO}*/ mir-274*^{KO}*; UAS-mir-274*	21	43.3 ± 2.31
mir-274^{KO}*/ mir-274*^{KO}*; elav>mir-274*	14	53.2 ± 4.39
mir-274^{KO}*/ mir-274*^{KO}*; 2x UAS-mir-274*	16	41.9 ± 2.4
mir-274^{KO}*/ mir-274*^{KO}*; elav> 2x mir-274*	16	64.9 ± 2.8
**4H (Trachea); right panel**		
**Genotypes**	**n**	**Mean ± SEM**
mir-274^{KO}*/ mir-274*^{KO}*; elav-GAL4*	15	25.5 ± 1.13
mir-274^{KO}*/mir-274*^{KO}*; UAS-mir-274*	21	24.7 ± 0.988
mir-274^{KO}*/ mir-274*^{KO}*; elav>mir-274*	16	26.3 ± 1.11
mir-274^{KO}*/ mir-274*^{KO}*; 2x UAS-mir-274*	13	28.1 ± 2.62
mir-274^{KO}*/mir-274*^{KO}*; elav>2x mir-274*	13	27.8 ± 1.78

**4J (Boutons); left panel**

Genotypes	n	Mean ± SEM
mir-274^{KO}/ mir-274^{KO}; btl-GAL4	20	45 ± 1.66
mir-274^{KO}/ mir-274^{KO}; UAS-mir-274	19	43.8 ± 2.77
mir-274^{KO}/ mir-274^{KO}; btl>mir-274	10	40.4 ± 2.18
mir-274^{KO}/mir-274^{KO}; 2x UAS-mir-274	18	43.6 ± 2.24
mir-274^{KO}/mir-274^{KO}; btl>2x mir-274	16	45.8 ± 3.07

**4J (Trachea); right panel**		
**Genotypes**	**n**	**Mean ± SEM**
mir-274^{KO}/mir-274^{KO}; btl-GAL4	18	23.8 ± 1.51
mir-274^{KO}/mir-274^{KO}; UAS-mir-274	20	25.6 ± 1.43
mir-274^{KO}/mir-274^{KO}; btl>mir-274	12	27.4 ± 1.47
mir-274^{KO}/mir-274^{KO}; 2x UAS-mir-274	14	32.6 ± 1.86
mir-274^{KO}/mir-274^{KO}; btl>2x mir-274	14	46.9 ± 2.54

Figure 5

**5B**		
**Genotypes**	**n**	**Mean ± SEM**
vector control	3	1 ± 0.108
vector with *sty* 3’UTR	3	0.656 ± 0.0145
vector with mismatch *sty* 3’UTR	3	1.44 ± 0.0505

**5C**		
**Genotypes**	**n**	**Mean ± SEM**
w^{1118} (1, n = 3)	3	1
mir-274^{KO}/mir-274^{KO} (, n = 3)	3	1.377 ± 0.1385

**5E (Boutons); left panel**		
**Genotypes**	**n**	**Mean ± SEM**
w^{1118}	12	1 ± 0.144
mir-274^{KO}/mir-274^{KO}	12	1.8 ± 0.333
**5E (Trachea); right panel**		
**Genotypes**	**n**	**Mean ± SEM**
w^{1118}	15	1 ± 0.106

mir-274^{KO}/*mir-274*^{KO}	14	1.71 ± 0.142

**5G (Boutons); left panel**		
**Genotypes**	**n**	**Mean ± SEM**
w^{1118}	27	1 ± 0.0313
mir-274^{KO}/*mir-274*^{KO}	16	0.661 ± 0.0533
mir-274^{KO}/*mir-274*^{KO}; *sty*^{226}/+	12	1.57 ± 0.233
mir-274^{KO}/*mir-274*^{KO}; *sty*$^{\Delta 5}$/+	20	1.28 ± 0.0665
**5G (Trachea); right panel**		
**Genotypes**	**n**	**Mean ± SEM**
w^{1118}	32	1 ± 0.0713
mir-274^{KO}/*mir-274*^{KO}	11	0.547 ± 0.0457
mir-274^{KO}/*mir-274*^{KO}; *sty*^{226}/+	11	1.18 ± 0.0946
mir-274^{KO}/*mir-274*^{KO}; *sty*$^{\Delta 5}$/+	18	1.02 ± 0.108

**5I (Boutons); left panel**		
**Genotypes**	**n**	**Mean ± SEM**
w^{1118}	12	60.3 ± 2.55
mir-274^{KO}/*mir-274*^{KO}	18	43.6 ± 2.72
mir-274^{KO}/*mir-274*^{KO}; *sty*^{226}/+	13	60.7 ± 2.99
mir-274^{KO}/*mir-274*^{KO}; *sty*$^{\Delta 5}$/+	13	60.5 ± 2.54
**5I (Trachea); right panel**		
**Genotypes**	**n**	**Mean ± SEM**
w^{1118}	11	36.7 ± 1.98
mir-274^{KO}/*mir-274*^{KO}	14	23.4 ± 1.63
mir-274^{KO}/*mir-274*^{KO}; *sty*^{226}/+	10	32.8 ± 1.67
mir-274^{KO}/*mir-274*^{KO}; *sty*$^{\Delta 5}$/+	16	32.3 ± 1.17

Figure 6

**6C (Boutons/Sty); left panel**		
**Genotypes**	**n**	**Mean ± SEM**
mir-274^{KO}/*mir-274*^{KO}; *repo-GAL4*	14	1 ± 0.101

mir-274^{KO}/*mir-274*^{KO}; *repo>mir-274*	16	0.457 ± 0.0166
**6C (Boutons/dpERK); left panel**		
**Genotypes**	**n**	**Mean ± SEM**
mir-274^{KO}/*mir-274*^{KO}; *repo-GAL4*	10	1 ± 0.11
mir-274^{KO}/*mir-274*^{KO}; *repo>mir-274*	10	1.31 ± 0.0738
**6C (Trachea/Sty); right panel**		
**Genotypes**	**n**	**Mean ± SEM**
mir-274^{KO}/*mir-274*^{KO}; *repo-GAL4*	24	1 ± 0.0996
mir-274^{KO}/*mir-274*^{KO}; *repo>mir-274*	24	0.523 ± 0.0442
**6C (Trachea/dpERK); right panel**		
**Genotypes**	**n**	**Mean ± SEM**
mir-274^{KO}/*mir-274*^{KO}; *repo-GAL4*	10	1 ± 0.0883
mir-274^{KO}/*mir-274*^{KO}; *repo>mir-274*	15	1.4 ± 0.107

**6F (Boutons); left panel**		
**Genotypes**	**n**	**Mean ± SEM**
mir-274^{KO}/*mir-274*^{KO}; *elav-GAL4*	16	41.3 ± 2.56
mir-274^{KO}/*mir-274*^{KO}; *elav>sty*^{RNAi}	20	53.6 ± 2.11
**6F (Trachea); left panel**		
**Genotypes**	**n**	**Mean ± SEM**
mir-274^{KO}/*mir-274*^{KO}; *elav-GAL4*	16	23.8 ± 1.55
mir-274^{KO}/*mir-274*^{KO}; *elav>sty*^{RNAi}	12	20.6 ± 1.82
**6F (Boutons); right panel**		
**Genotypes**	**n**	**Mean ± SEM**
mir-274^{KO}/*mir-274*^{KO}; *btl-GAL4*	15	45.8 ± 2.07
mir-274^{KO}/*mir-274*^{KO}; *btl>sty*^{RNAi}	16	46.1 ± 2.33
**6F (Trachea); right panel**		
**Genotypes**	**n**	**Mean ± SEM**
mir-274^{KO}/*mir-274*^{KO}; *btl-GAL4*	20	21.1 ± 1.334
mir-274^{KO}/*mir-274*^{KO}; *btl>sty*^{RNAi}	8	30.63 ± 2.492

Figure 7

**7A (1% O_2); left panel**

Genotypes	Time	n	Mean ± SEM
w^{1118}	5	19	21.1 ± 3.05
	10	19	36.8 ± 3.98
	15	19	42.6 ± 4.25
Canton S	5	12	20 ± 3.89
	10	12	32.5 ± 5.66
	15	12	47.5 ± 6.41
mir-274^{KO}/*mir-274*^{KO}	5	23	46.1 ± 4.01
	10	23	62.2 ± 3.5
	15	23	58.7 ± 3.89
**7A (10% O_2); middle panel**			
**Genotypes**	**Time**	**n**	**Mean ± SEM**
w^{1118}	5	7	15.7 ± 4.81
	10	7	14.3 ± 5.28
	15	7	11.4 ± 4.59
Canton S	5	7	24.3 ± 5.28
	10	7	12.9 ± 3.6
	15	7	4.29 ± 2.02
mir-274^{KO}/*mir-274*^{KO}	5	8	35 ± 4.5
	10	8	27.5 ± 1.64
	15	8	21.3 ± 2.95
**7A (21% O_2); right panel**			
**Genotypes**	**Time**	**n**	**Mean ± SEM**
w^{1118}	5	9	2.22 ± 2.22
	10	9	2.22 ± 1.47
	15	9	2.22 ± 2.22
Canton S	5	8	2.5 ± 1.64
	10	8	2.5 ± 1.64
	15	8	2.5 ± 1.64
mir-274^{KO}/*mir-274*^{KO}	5	7	2.86 ± 1.84
	10	7	4.29 ± 2.02
	15	7	4.29 ± 2.02

**7B (1% O_2)**			
**Genotypes**	**Time**	**n**	**Mean ± SEM**
mir-274^{KO}/ mir-274^{KO}; repo-GAL4	5	10	53 ± 5.17
	10	10	66 ± 4.27
	15	10	73 ± 5.17
mir-274^{KO}/mir-274^{KO}; UAS-mir-274	5	13	45.4 ± 2.68
	10	13	64.6 ± 3.32
	15	13	73.8 ± 3.5
mir-274^{KO}/mir-274^{KO}; repo>mir-274	5	11	28.2 ± 3.77
	10	11	49.1 ± 4.76
	15	11	58.2 ± 2.96

**7C**			
**Genotypes**	**Time**	**n**	**Mean ± SEM**
mir-274^{KO}/+; repo-GAL4	10	10	37.5 ± 6.478
mir-274^{KO}/mir-274^{KO}; repo>decoy-mir-274	10	10	71.25 ± 2.266
mir-274^{KO}/+; elav-GAL4	10	9	38.89 ± 6.961
mir-274^{KO}/mir-274^{KO}; elav>decoy-mir-274	10	10	46 ± 5.207
mir-274^{KO}/+; btl-GAL4	10	7	25.71 ± 5.281
mir-274^{KO}/ mir-274^{KO}; btl>decoy-mir-274	10	9	65.56 ± 4.12

**7D (1% O_2)**			
**Genotypes**	**Time**	**n**	**Mean ± SEM**
w^{1118}	5	10	31 ± 3.79
	10	10	36 ± 2.67
	15	10	38 ± 4.67
mir-274^{KO}/ mir-274^{KO}	5	11	54.5 ± 5.62
	10	11	60.9 ± 2.85
	15	11	72.7 ± 4.07
mir-274^{KO}/mir-274^{KO}; sty^{226}/+	5	10	29 ± 6.57

	10	10	34 ± 6.36
	15	10	42 ± 3.27
mir-274^{KO}/mir-274^{KO}; sty$^{\Delta 5}$/+	5	10	35 ± 6.37
	10	10	39 ± 5.26
	15	10	41 ± 5.67

References

1. R. J. Bainton *et al.*, *moody* encodes two GPCRs that regulate cocaine behaviors and blood-brain barrier permeability in *Drosophila*. *Cell* **123**, 145-156 (2005).
2. S. Roy, H. Huang, S. Liu, T. B. Kornberg, Cytoneme-mediated contact-dependent transport of the *Drosophila* Decapentaplegic signaling protein. *Science* **343**, 1244624 (2014).
3. T. Haraguchi, Y. Ozaki, H. Iba, Vectors expressing efficient RNA decoys achieve the long-term suppression of specific microRNA activity in mammalian cells. *Nucleic Acids Res* **37**, e43 (2009).
4. N. Hacohen, S. Kramer, D. Sutherland, Y. Hiromi, M. A. Krasnow, *sprouty* encodes a novel antagonist of FGF signaling that patterns apical branching of the *Drosophila* airways. *Cell* **92**, 253-263 (1998).
5. C. H. Wang *et al.*, USP5/Leon deubiquitinase confines postsynaptic growth by maintaining ubiquitin homeostasis through Ubiquilin. *Elife* **6** (2017).
6. H. M. Kerstens, P. J. Poddighe, A. G. Hanselaar, A novel *in situ* hybridization signal amplification method based on the deposition of biotinylated tyramine. *J Histochem Cytochem* **43**, 347-352 (1995).
7. J. T. G. Pena *et al.*, miRNA *in situ* hybridization in formaldehyde and EDC-fixed tissues. *Nat Methods* **6**, 139-141 (2009).
8. S. G. Zimmerman, N. C. Peters, A. E. Altaras, C. A. Berg, Optimized RNA ISH, RNA FISH and protein-RNA double labeling (IF/FISH) in *Drosophila* ovaries. *Nat Protoc* **8**, 2158-2179 (2013).
9. C. I. Nussbaum-Krammer, M. F. Neto, R. M. Brielmann, J. S. Pedersen, R. I. Morimoto, Investigating the spreading and toxicity of prion-like proteins using the metazoan model organism *C. elegans*. *J Vis Exp* 10.3791/52321, 52321 (2015).
10. J. A. Wingrove, P. H. O'Farrell, Nitric oxide contributes to behavioral, cellular, and developmental responses to low oxygen in *Drosophila*. *Cell* **98**, 105-114 (1999).
11. D. L. Brink, M. Gilbert, X. Xie, L. Petley-Ragan, V. J. Auld, Glial processes at the *Drosophila* larval neuromuscular junction match synaptic growth. *PLoS One* **7**, e37876 (2012).
